# Exploring the bidirectional temporal association between daily knee pain and physical activity in people with knee osteoarthritis: An exploratory smartwatch study

**DOI:** 10.1016/j.ocarto.2026.100753

**Published:** 2026-01-31

**Authors:** Ayobami E. Olanrewaju, Matthew J. Parkes, Jamie C. Sergeant, Emma Pritchard, Shuai Shao, Stephanie R. Filbay, Sabine N. van der Veer, David Wong, William G. Dixon

**Affiliations:** a Division of Informatics, Imaging and Data Sciences, School of Health Sciences, University of Manchester, United Kingdom; b Centre for Biostatistics, Division of Population Health, Health Services Research and Primary Care, School of Health Sciences, University of Manchester, United Kingdom; c Centre for Health, Exercise and Sports Medicine, Department of Physiotherapy, University of Melbourne, United Kingdom; d Leeds Institute of Health Science, University of Leeds, United Kingdom; e NIHR Manchester Biomedical Research Centre, Manchester University NHS Foundation Trust, Manchester Academic Health Science Centre (MAHSC), United Kingdom

**Keywords:** Physical activity, Knee osteoarthritis, Knee pain

## Abstract

**Objective:**

Effective physical activity interventions for knee osteoarthritis (OA) require an understanding of the relationship between physical activity and pain. Using daily concurrent activity and pain measurements, we explored day-to-day changes and the bidirectional temporal association at individual and population level.

**Design:**

This is a secondary analysis of step count and pain collected for 90 days using smartwatches in 26 people with knee OA. People reported pain twice daily on a Numerical Rating Scale (NRS 0–10). We used regression (individual level) and generalized linear mixed models (population) to explore same-day associations, as well as whether step count on one day predicted pain the next day, and vice versa.

**Results:**

We analysed 1473 daily pain and step count measurements, recorded over a median 58 days. There were considerable day-to-day changes in individuals’ median step count (range 423–7142) and pain (range 0–9). At individual level, associations varied in the strength and direction. At population level, a higher step count was associated with higher pain on the same day (0.04 NRS/1000 step increase, 95%CI 0.01–0.06) and following day (0.05/1000 step increase, 0.03–0.07).

**Conclusions:**

There was a modest association at population level between step count assessed on one day and pain assessed on the same and following day. However, there was variation in the strength and direction of associations when examined at the individual level. This exploratory analysis shows how smartwatches allow daily data collection that enables detailed exploration of complex time-varying relationships in OA.

## 1 Introduction

Knee osteoarthritis (OA) affects over one in three people in their lifetime [1] and causes pain and impairment in physical activity (PA) [2], both of which vary over time. The exact causal relationship between pain and PA is unclear, but is likely complex and reciprocal. Pain may limit PA [3,4] by altering how an activity is performed or by leading to avoidance [5] to alleviate discomfort [6]. Conversely, PA may increase pain during activity, and for some time afterwards [7]. Both effects may occur simultaneously, where pain and activity interact in a complex way over time. Understanding the temporal relationship between pain and PA may help develop future outcome measures for knee OA and may help inform the development of future PA-focused interventions designed to reduce pain in knee OA [8].

Knowing the relationship between pain and PA requires accurate, daily concurrent measurement, since both can change within a day and between days [[9], [10], [11]]. Some studies seeking to explore the relationship between pain and PA have used sparse or infrequent measures, often measured at different time points (e.g. measurements taken days apart), thus leaving their association unclear [4,[12], [13], [14], [15]]. In studies that did collect pain and PA on the same day, a positive relationship was found whereby more time spent in moderate to vigorous PA was associated with increased pain on the same day [9] and time spent exercising [10] was associated with pain recorded on the same day. Although both studies measured pain and PA on the same day, neither study examined how these varied over a period greater than 2-weeks, nor whether the relationship seen within the participants was the same as the relationship observed across the study population. Collecting data over a longer duration may offer a clearer understanding of the relationship between pain and PA, for example by capturing less common flares in OA pain [16] or days with particularly high PA. In addition, knowing about within-person associations may help plan personalised interventions that are more appropriate to individual needs, which is noted as a challenge to effective PA intervention strategies [17].

Frequent (e.g. daily) measurement of time-varying pain and PA requires us to consider how this might be feasibly measured in individuals. Self-reported information is typically regarded as low-cost and easy to do [18], but measuring daily is potentially burdensome. Reporting daily symptoms via paper diaries requires study participants to remember to record, and to have their diary to hand if-and-when they do remember. Recall of PA using questionnaires is notoriously inaccurate [19,20] and carries a similar burden. Consumer technology like smartwatches have the potential to overcome the limitations of self-reported PA because devices have sensors that continuously and passively collect PA, while their touchscreen allows active data collection. For instance, data such as pain scores can be obtained at the time pain occurs, which decreases the recall ‘window’, and hence may reduce the likelihood of recall bias. Likewise, this approach makes active data collection to be more seamlessly integrated into people's lives.

The Knee OsteoArthritis Linking Activity with Pain (KOALAP) study was a smartwatch feasibility study that aimed to measure pain and PA daily for 90 days in 26 people living with knee OA. A previous study using the KOALAP data showed that pain categories and PA could vary across individuals, and activity level accumulation may depend on pain variation category [11]. However, that study did not address the temporal association between pain and PA. This current study aims to: (1) describe the day-to-day changes in pain and PA [measured as a step count] in people with knee OA, and (2) investigate the bidirectional temporal association of pain and PA both at an individual level and across the population. The bidirectional association considers associations between pain and PA (and vice versa) on the current day, associations between their current-day and prior-day values, and associations between their current-day values and changes from the prior day to the current day.

## 2 Method

### 2.1 Design and participants

This is a secondary analysis using data from the KOALAP study. KOALAP was an observational, feasibility study that investigated day-to-day variation in pain and activity in people with self-reported knee OA. Further details are available in the study protocol [21] and analysis of participant engagement [22]. The KOALAP study (parent study) included individuals aged 50 and older that had self-reported knee OA, who either lived in Greater Manchester or were willing to travel to Manchester [21]. The self-reported diagnosis was not validated by a clinician. Smartwatches (Huawei Watch 2) were provided to participants for the duration of the study. Participants' pain scores were collected in the afternoon and the evening from a bespoke app displayed on the smartwatch screen, and step count was continuously measured using the smartwatch's sensors and derived using the proprietary device algorithm over a 90-day period. The study was conducted from September 2017 to January 2018. All participants provided informed consent, and the study was approved by the University of Manchester Research Ethics Committee (#0165). The present analysis received University Research Ethics Committee approval (Reference: 2024-21422-37727). KOALAP participants were included in this secondary analysis if they had at least two days of pain reports and step count records. In the primary feasibility study, 20 people was the minimum required sample size when potential attrition was considered [21].

### 2.2 Physical activity monitoring

The daily estimated step count was used as the PA measure for this study's analysis. Participants were asked to wear their smartwatch from waking until bedtime. Step count was generated using the smartwatch's algorithm. Further details can be found in Vivekanantham et al., 2023 [11].

### 2.3 Daily knee pain

During the set-up of the device, each participant identified the knee (left or right) most affected by OA and were instructed to answer all study questions with that pre-selected knee in mind. Participants were prompted via vibrating notification to report their level of knee pain twice daily using the prompt “Level of knee pain”: Prompts occurred in the afternoon at 12:22pm (completion window of 12:22pm–4pm) and in the evening at 6:22pm (completion window of 6:22pm–10pm). This procedure was repeated daily for 90 days using the study app displayed on the smartwatch's screen. The level of knee pain was reported on a 0–10 numerical rating scale (NRS) [0 = no pain at all; 10 = extreme pain]. The level of pain response was regarded as missing if participants did not respond within the above 4-h time windows.

### 2.4 Other measures

Demographic information (age, sex, body mass index [BMI]) was obtained from the participants during enrolment. Participants completed the Knee Injury and Osteoarthritis Outcome Score (KOOS) pain and activities of daily living subscales within the first 2–3 weeks following enrolment.

### 2.5 Handling missing data

This study used a complete observation analysis approach and did not impute missing values.

### 2.6 Data analysis

#### 2.6.1 Data preparation

Each participant contributed data from their first day of data entry to 90 days, with follow-up censored at 90 days. Participants' data was included in the analysis when both pain and step count were available for any given day. Daily pain was taken as a mean of the afternoon and evening pain if both were available for that day. If one of either afternoon or evening pain was missing, the remaining pain score was used. Pain was collapsed into a single measure as a proxy for daily pain to match daily step count records, and all available pain reports were used to maximize the number of data points. If pain was not reported on a given day at either time point, that day did not contribute to the analysis.

### 2.7 Analysis methods

For the first aim of describing day-to-day changes in pain and PA, the distribution of median (interquartile range) and range of the mean daily pain reports and step count across days for all individuals were reported. The day-to-day time-varying daily pain reports and step count was plotted for each individual. Examples of individuals’ pain and PA through time were presented in the results to illustrate what the time-series data looks like, for both an individual where an association seems present and for someone where it is less clear. All other participant-level plots were included in the supplementary file 1.

For the second aim of investigating the bidirectional temporal association of daily pain and PA at an individual level and across the population, the association was assessed under five association analyses. Using the following annotations and defining “current day” as day t, the bidirectional temporal association were assessed as the associations between:

(1) Current day's pain [day t] and current day's step count [day t].
(2) Current day's pain [day t] and prior day's step count [day t −1] (hypothesis: higher activity on the prior day is associated with an increase in current day's pain).
(3) Current day's pain [day t] and change from prior day's to the current day's step count [(day t) – (day t – 1)] (hypothesis: increase in activity from prior to current day is associated with an increase in current day pain).
(4) Current day's step count [day t] and prior day's pain [day t −1] (hypothesis: higher pain on the prior day is associated with a reduction in current day activity).
(5) Current day's step count [day t] and change from prior day's to the current day's pain [(day t) – (day t – 1)] (hypothesis: increase in pain from prior to current day is associated with a reduction in current day activity).

Ordinary least squares (OLS) regression was used to model the association between daily pain and step count within each participant. The within-person analyses results (estimate and confidence interval [CI]) obtained from the OLS regression were plotted for all participants for the above five association analyses, with participants ranked by the magnitude of the association.

A generalized linear mixed (GLM) effect model was used to determine the association between daily pain and step count between participants (population-wide analysis). The model took the daily pain and step count variant as the outcome or fixed effect. Participant identifier was the random effect to account for variation in each participant's data. The mixed-effects model accommodates differences in the number of observations across participants by allowing each participant to contribute data for all days on which they have measurements [23,24]. It does not weight participants differently based on observation count; however, under maximum likelihood estimation, participants with more observations contribute more information to the model fit. The population-wide estimate and CI generated using the GLM model were appended to the within-individual association plot as the right-most data points on each association plot. The population-wide estimates and CI were also presented in a table for all five association analyses.

All data analysis was conducted using R version 4.4.3 using the R libraries ggplot2[], patchwork[], dplyr[], ggbreak[], lmer[][a GLM model – a function from the lmer4 library] and lm[][linear regression model] (see supplementary file 2 for all analysis codes).

### 2.8 Sensitivity analyses

Three sensitivity analyses were conducted to assess the robustness of the findings.

1) The analysis examining association between daily pain [day t] and step count [day t] was repeated using afternoon pain, evening pain and the mean of afternoon and evening pain when both were present.
2) The analysis for association of daily pain [day t] and step count [day t] was repeated using data from participants with more than 20 % and more than 50 % of the 90-day observation period available.
3) The five analyses examining bidirectional association between daily pain and step count were adjusted first for age, sex, BMI, then additionally for day of the week. These adjustments were applied only to population-level analyses, as these potential confounder (age, sex, BMI) are largely time invariant and therefore not relevant to a within-person analysis.

## 3 Results

All 26 participants (i.e., the full sample) recruited into the KOALAP study were included in all analyses. Participants had a median [IQR] age of 64 [59–69] years, included an equal number of men and women, and had a median [IQR] body mass index of 27 [[25], [26], [27], [28], [29], [30], [31], [32], [33], [34]] (Table 1).

**Table 1:** Demographics characteristics of KOALAP participants [n = 26].

Age, [years], Median [IQR]	64 [59–69]
BMI, kg/m^2^, median [IQR]	27 [[25], [26], [27], [28], [29], [30], [31], [32], [33], [34]]
Sex, n [%]	
Female	13 [50]
Male	13 [50]
KOOS, median [IQR]	
aPain subscale	48.6 [41.0–59.0]
bActivity of daily living subscale	55.9 [42.3–68.4]

N: number; IQR: interquartile range; SD: standard deviation; KOOS: Knee Injury and Osteoarthritis Outcome Score.

a This is based on the score for question 1 to 9 of the KOOS questionnaire's pain subscale, obtained in the first month of data collection.

b This is based on the score for questions 1 to 17 of the KOOS questionnaire's Activities of Daily Living subscale, obtained during the first month of data collection.

Collectively they contributed a median of 58 days (range 7–90 days) where both pain and step counts were reported on the same day. Eighteen (69 %) of all participants had more than 50 days’ data. In total, there were 1473 daily pain reports and step count records out of a potential 2340 records (90 × 26), equating to 63 % of possible days with complete data. All participants contributed data to the analyses for association analysis 2-5 that required complete data for adjacent days [e.g. t-1 to t], with a median of 51 days (range 2–89 days). In total, there were 1263 adjacent daily pain reports and step counts out of a potential 2314 records (89 × 26). The median[IQR] device daily wear time among participants was 11 h 12 min [9 h 27 min - 12 h 6 min] [22].

### 3.1 Day-to-day changes in pain and step count in people living with knee OA

The median [IQR] daily pain per participant was 5 [[2], [3], [4], [5], [6], [7]] [range 0–9], and the median [IQR] daily step count was 3106 [1687–4433] [range 423–7142]. Within individuals across the 90 days, there was notable day-to-day variation in the distribution of pain and step count. Using two illustrative examples, Fig. 1 shows how pain and step count changed for one participant, with an apparent visual correlation between increases in step count and increases in pain. Fig. 2 illustrates a different participant, where the correlation between step count and pain was less visually apparent. The full set of pain and step count plots for all the remaining participants is provided in supplementary file 1 (Section A, Figs. 1–24).

**Fig. 1:**
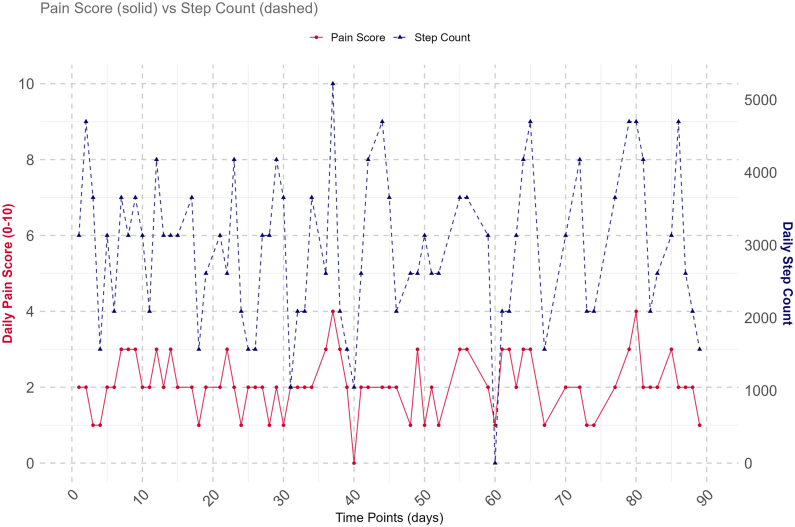
Daily pain and step count over time for Participant 21.

**Fig. 2:**
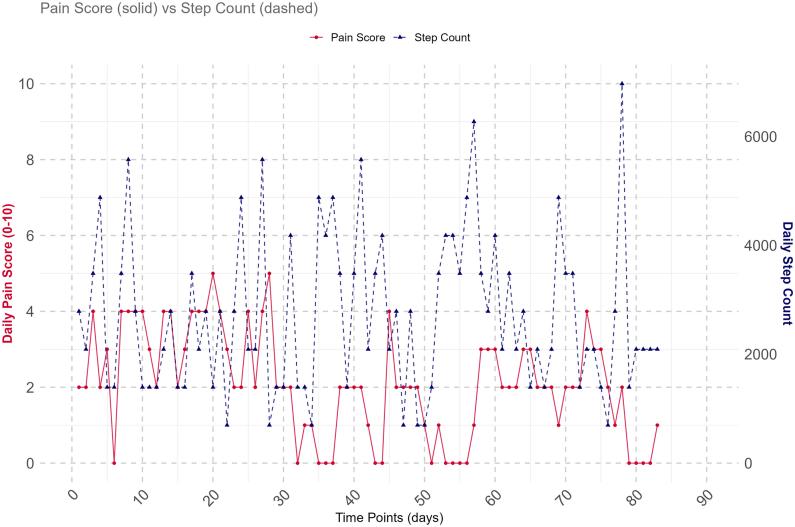
Daily pain and step count over time for Participant 30.

### 3.2 Bidirectional association between pain and physical activity

#### 3.2.1 Associations within individuals

**Fig. 3:**
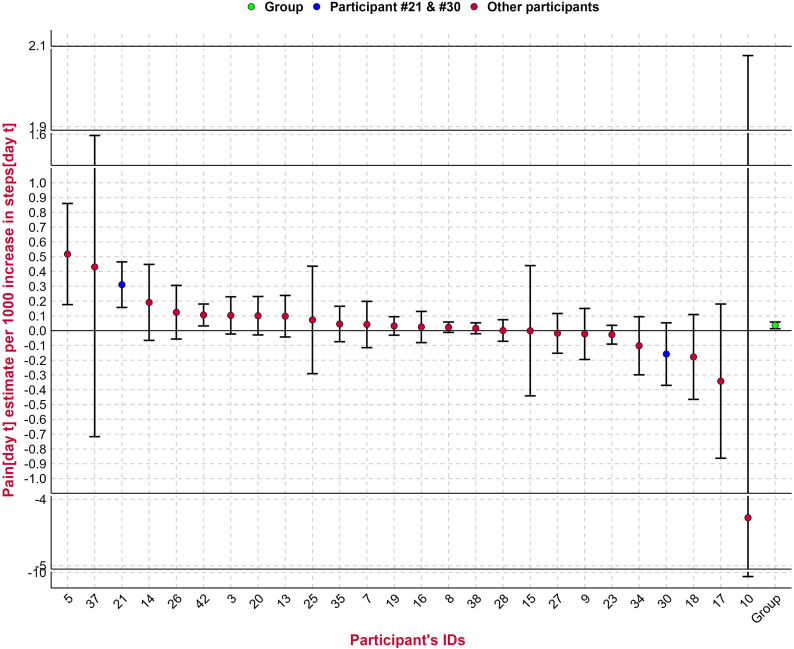
Association between pain and step count [per 1000 unit increases in step count] on the current day [day t] for each participant plus whole population [“Group”] estimate. Each participant is represented within the plot, ranked by their strength of association, with the vertical lines representing confidence intervals. Participants 10 and 37 had wide confidence intervals due to sparse data, requiring an illustration using a break in the plot. The point estimates coloured blue highlight participants 21 and 30 whose pain and step count distributions are shown in both Fig. 1, Fig. 2, illustrating where they lie in the population-wide distributions for the analysis. The green point estimate on the right represents the whole population, or group, estimate which is also reported in Table 2.

**Fig. 4:**
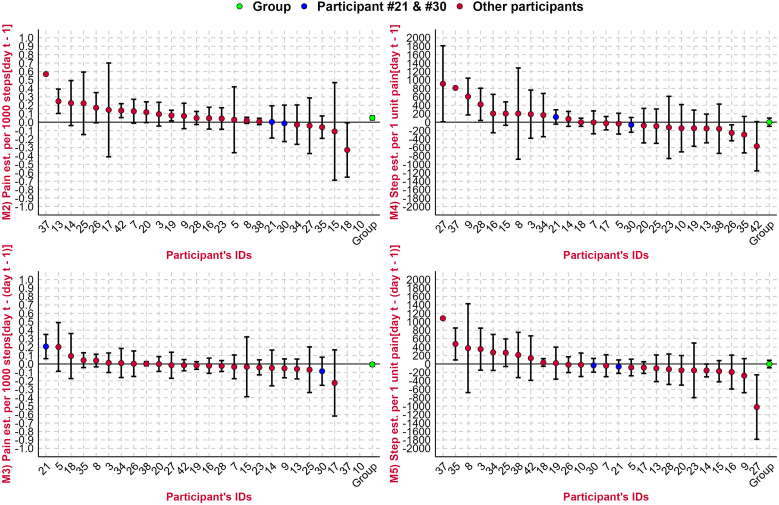
Association between pain and step count [per 1000 unit increase in step count], or step count and pain [per 1 unit increase in NRS pain] for each participant plus whole population [“Group”] estimate. The preceding M is a short form for Model 2 to 5 in Table 2. M2) = the current day's pain [day t] and prior day's step count [day t - 1]; M3) = the current day's pain [day t] and change from prior day's to the current day's step count [(day t) – (day t – 1)]; M4) = the current day's step count [day t] and the prior day's pain [day t - 1]; M5) = current day's step count [day t] and change from prior day's to the current day's pain [(day t) – (day t – 1)]. est. = estimate. Day t = current day. Each participant is represented within the plot, ranked by their strength of association, with the vertical lines representing confidence intervals. Participants 10 and 37 had wide confidence intervals due to sparse data, so their estimate and CI could be missing due to difficulty to fit them into Fig. 4, or they are unavailable; See supplementary file 1 [Section B, Figure B1 to B4] for independent Figures for M2 to M5. The point estimates coloured blue highlight participants 21 and 30 whose pain and step count distributions are shown in both Fig. 1, Fig. 2, illustrating where they lie in the population-wide distributions for the different analyses. The green point estimate on the right represents the whole population, or the group, estimate which is also reported for Model 2 to 5 on Table 2.

Three participants (#5, #21 and #42) had a statistically significant positive association between pain and step count measured on the current day [day t] (Fig. 3). Three participants (#13, #19, and #42) also had a statistically significant positive association between the current day's pain [day t] and the prior day's step count [day t-1], with one participant (#18) having a statistically significant negative association (Panel M2, Fig. 4). Only one participant (#42) had a positive association for both analyses. The remaining three analyses (current day's pain and change in steps, current day's steps and prior day's pain, and current day's steps and change in pain) showed a similar pattern of varying results across the population of participants, sometimes including statistically significant results at the extremes in both positive and negative directions.

#### 3.2.2 Associations across the population

Across all participants, there was a modest overall positive association between pain [day t] and step count [day t], whereby higher step count was associated with higher pain [0.04 NRS pain per 1000 unit increase in steps, 95 % CI 0.01 to 0.06]. The modest positive association between pain [day t] and step count [day t] remained consistent even when pain was measured using evening pain and the average of afternoon and evening pain (supplementary file 3) and when analyses were restricted to participants with more than 20 % or more than 50 % of observations available (supplementary file 4). However, this association was attenuated when using afternoon pain. There was also a positive association between the current day's pain [day t] and the prior day's step count [t-1] [0.05 NRS pain per 1000 unit increase in steps, CI 0.03 to 0.07] (Table 2). There was no observed association for the remaining analyses (current day's pain and change in steps, current day's steps and prior day's pain, and current day's steps and change in pain). The results were similar when adjusted for age, sex, BMI and day of the week (supplementary file 5).

**Table 2:** Associations between pain and step count, or step count and pain across all 26 participants.

Model	Outcome	Independent Variable	Estimate [95 % CI]	Effect size [95 % CI]	p-value
Model 1	Current day pain [day t]	Current day step count [day t]	0.04 [0.01 to 0.06]	0.05 [0.02 to 0.08]	0.002
Model 2	Current day's pain [day t]	Prior day's step count [day t-1]	0.05 [0.03 to 0.07]	0.07 [0.04 to 0.11]	<0.001
Model 3	Current day's pain [day t]	Change from prior day's to the current day's step count [(day t) – (day t-1)]	−0.01 [-0.03 to 0.01]	−0.01 [-0.04 to 0.02]	0.544
Model 4	Current day's step count [day t]	Prior day's pain [day t – 1]	−0.4 [-98 to 97]	−0.0002 [-0.05 to 0.05]	0.994
Model 5	Current day's step count [day t]	Change from prior day's to the current day's pain [(day t) – (day t-1)]	−2 [-90 to 86]	−0.001 [-0.05 to 0.04]	0.960

Associations reported are per 1000 unit increase in step count, or per one unit increase in NRS pain, depending on the analysis. ‘day t’ represents current day. CI: Confidence Interval. Effect size = standardized effect size. Effect size is computed by multiplying the model estimate by the ratio of the standard deviation of the predictor to the standard deviation of the outcome. It indicates how many standard deviations the outcome increases or decreases for a one-standard-deviation increase (or decrease) in the predictor.

## 4 Discussion

This exploratory analysis examined the association between time-varying pain and step count for 26 people living with knee OA. Across all participants, higher step counts were associated with an increase in knee pain within the current day [day t]. There was also an observed positive association between the current day's pain [day t] and the prior day's step count [day t-1], but no association between current day's pain and change in steps from the prior day to the current day, current day's steps and prior day's pain, and current day's steps and change in pain from the prior day to the current day. Where associations were present, the average population effect sizes were modest. The positive but small association between current-day pain and step count would mean a one-unit increase in pain, corresponding to the minimal clinically important difference for a ten-point numerical rating scale associated with patient improvement [25] and extrapolated here as a useful metric to anchor our findings, would be associated with an increase of approximately 25,000 steps.

Individual participants varied in their observed associations between pain and step count. Although many had null associations statistically, some had associations that were significant and appeared clinically meaningful. For example, one of our within-person analysis showed that an increase in pain by one-unit would be associated with an increase of 2000 steps, which is well within observed day-to-day differences in step count. Where significant associations did exist, this was not consistently in one direction. Participants also appeared to demonstrate different strengths of association across analyses, illustrated by their difference in participants ranking based on their point estimates. This diverse range of patterns within and between individuals may reflect true heterogeneity within the population: something that would have been lost had we generated only population-wide estimates. A longer follow-up period and a larger number of participants might enable a clearer understanding of the observed wide intra- and inter-individual variability.

While causal inferences cannot be made, our finding suggests the association may lean toward higher step counts preceding higher pain, rather than pain influencing activity at the population level. This finding supports findings from other studies. Robbin et al. 2011 [26] shows that doing more activity leads to more loading of the knee, thereby leading to increased pain. In contrast, Kim et al. 2025 [27] found that being more sensitive to pain was associated with lower joint loading during walking. Considering people with knee OA are encouraged to embrace an active lifestyle understanding the ways to manage their pain is vital to sustainable active lifestyle adoption. Lee et al. 2015 [28], and Dawson et al. 2024 [29] found that people with knee OA spend two-thirds of their daily lives being sedentary. Being sedentary increases the risk of chronic disease, while an active lifestyle reduces functional limitations [30,31], improves bone health [32,33] and may help with pain [34,35]. Understanding how PA influences pain for a given individual, it is possible to imagine personalised activity coaching where some PA is encouraged, but staying within a limit of activity that would not significantly adversely impact pain for that person.

Most previous studies examining the relationship between knee pain and PA were largely cross-sectional designs and captured pain and activity at different time points which may have limited their ability to characterise the relationship. Within those studies they found no association between pain and PA measure [4,[12], [13], [14], [15]]. Those that captured pain and activity at almost the same time found some association between both [6,9,10,36]. For example, Chang et al. 2025 [9] using 10 days data from 17 people found that more moderate to vigorous PA was associated with more pain, while light PA was not associated with pain within the same day. However, neither measure of PA intensity was associated with the next day's pain. This may suggest that pain and activity may have complex temporal associations as we found in this current study. The magnitude of the association found in this study [9] was also similar to ours. It is important to note that several factors may impact the direction and magnitude of association that exist between pain and PA, such as knee OA severity [37], the activity type [37] and intensity of activity performed [38], whether the activity is ongoing or how recently it was completed [7], and individual differences in pain tolerance [39]. This may in part account for some of the between-person differences seen amongst our participants. Although our work could not account for these factors because they were not collected, it extends the work of prior studies by collecting daily concurrent pain and step counts for a longer period [90 days] and examining the association on a population level and as well on the participant level.

While this preliminary analysis could lay a foundation for teasing out the pain and PA relationships, our study has some limitations. The small sample size limits generalisation to a wider population. Despite the small numbers, capturing data for 90 days for each participant provided over 1000 observations which allowed us to investigate the association in our self-selected population. Next, we only had access to daily measures of step count which makes it difficult to know how the step count is accumulated through the day and how that affects the pain. An exploratory analysis of afternoon and evening pain [not reported] showed that people reported more pain in the evening than in the afternoon. This could suggest that pain reporting is influenced by all activity done through the day, and which would not be captured by a daily summary. Also, we did not consider how other factors such as medications, environmental factors, or psychosocial factors may have influenced the relationship between pain and activity. In addition, we used a bespoke app to report pain that had a radial numerical rating scale on the circular watch's screen [21]. This has not been validated, but has face validity and was well understood by our participants [22]. Step count, our main PA measure, is well validated across different devices [40]. The smartwatch used in this study has not been specifically validated for measuring step count in people with knee OA; however similar smartwatch devices have demonstrated sufficient validity for assessing step count in this population [41], and our estimated step count was assumed to be comparable to those obtained from other validated devices. Lastly, the population within the study self-reported their knee OA status and there could be variation in severity level. Nonetheless, the participants' age, step count and reported pain levels were within range of other similar studies [9,13,26,36] and we were interested in within-person change and the association between the two time-varying symptoms of knee OA.

In conclusion, in this exploratory study of people living with knee OA, step count and pain both varied from day-to-day. Individual participants would sometimes show an association between pain and steps, although often in different patterns across individuals. Only modest associations were seen across the population, representing a pooling of the wide variety of positive, null and negative effect sizes within participants. Significant findings were seen in the associations between higher step count with increased knee pain on the same day, and higher prior day's step count and increased pain on the following day. Although caution should be exercised when interpreting our findings due to the exploratory nature of the study, the analysis illustrates the complex relationship between pain and PA, both within and between individuals, and how both symptoms of knee OA change significantly through time. It also shows how consumer technology allows us to collect data that enable a more detailed exploration of this complex relationship. Therefore, pointing to a future where a more accurate measurement of pain and PA through time could support better outcome measures for knee OA or support personalised interventions.

## Author contributions

AEO is a PhD candidate, and this research forms part of her doctoral dissertation. MJP, JCS, and WGD were involved in the collection and assembly of data for the study. AEO, MJP, JCS, SRF, WGD were involved in the conception and design of the study. AEO was involved in the data analysis and all authors (AEO, MJP, JCS, EP, SS, SRF, SNV, DW, WGD) were involved in the data interpretation. AEO drafted the article and got critical revisions for intellectual content from all authors (MJP, JCS, EP, SS, SRF, SNV, DW, WGD). MJP, JCS, EP and DW provided statistical expertise. All authors (AEO, MJP, JCS, EP, SS, SRF, SNV, DW, WGD) approved the final version of the article. WGD and AEO are responsible for the integrity of the work.

## Role of the funding source

No dedicated funding was used to support this current study. However, the main KOALAP study was funded by Versus Arthritis [grants 21225 and 21755].

AEO is supported by the joint-PhD award program scholarship from the University of Manchester and the University of Melbourne. SRF is supported by a National Health and Medical Research Council (NHMRC) Investigator Grant [#1194428].

MJP and WGD are supported by the National Institute for Health and Care Research (NIHR) Manchester Biomedical Research Centre [NIHR203308]. The views expressed are those of the author[s] (MJP and WGD) and not necessarily those of the National Institute for Health and Care Research or the Department of Health and Social Care.

## Conflict of interest

All authors have no conflicts of interest to declare.
