# Supplementary material for: Exploring the bidirectional temporal association between daily knee pain and physical activity in people with knee osteoarthritis: An exploratory smartwatch study
Source: Osteoarthr Cartil Open. 2026 Jan 31;8(1):100753. doi: 10.1016/j.ocarto.2026.100753 (PMC12907850; doi:10.1016/j.ocarto.2026.100753)
Supplement: Multimedia component 1 [file mmc1.docx]

**Supplementary file 1 to the article** “Exploring the bidirectional temporal association between daily knee pain and physical activity in people with knee osteoarthritis: an exploratory smartwatch study.”

**Section A:** Daily pain and step count over time for the remaining 24 KOALAP study participants.


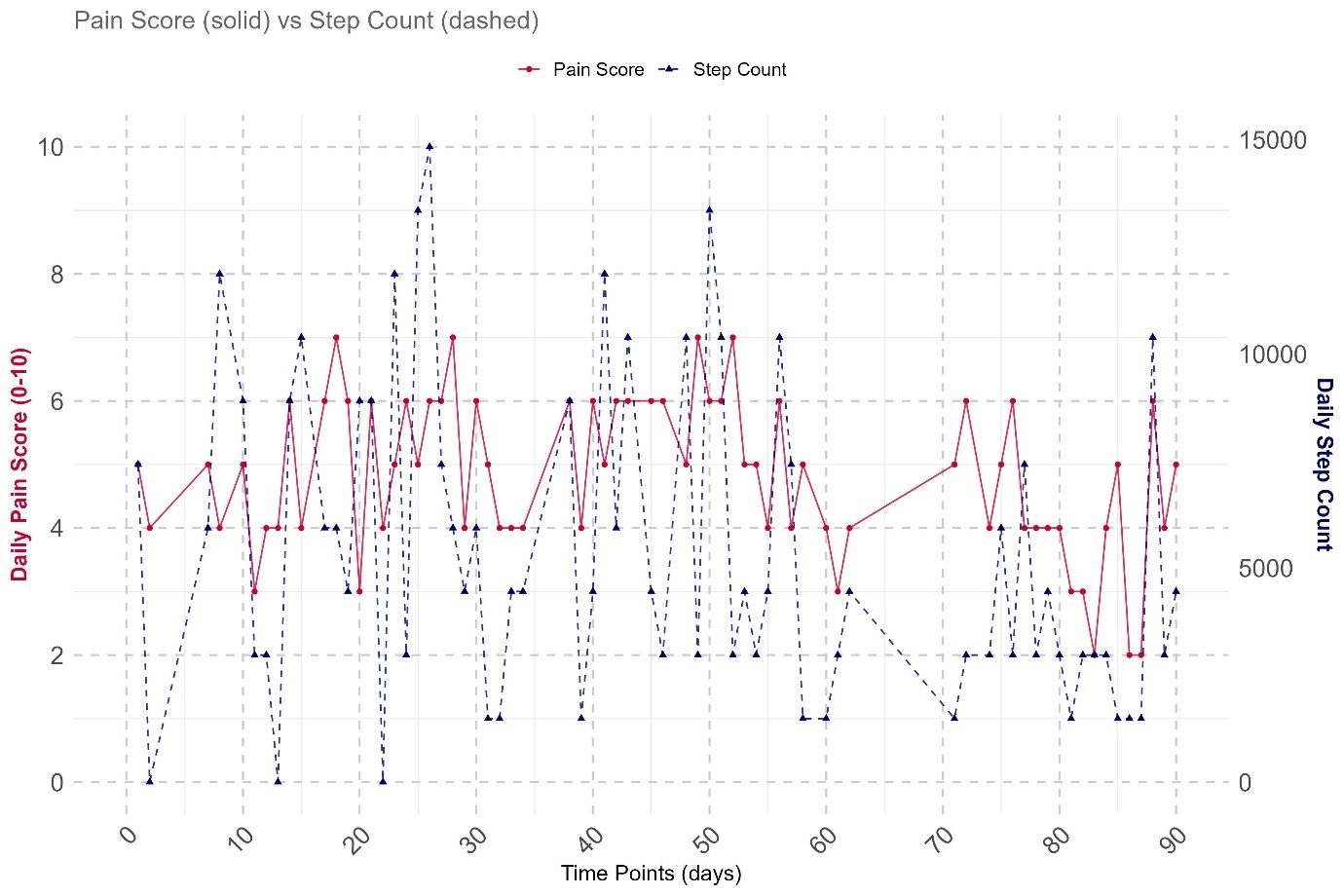


Figure 1: Daily pain and step count over time for Participant 42.


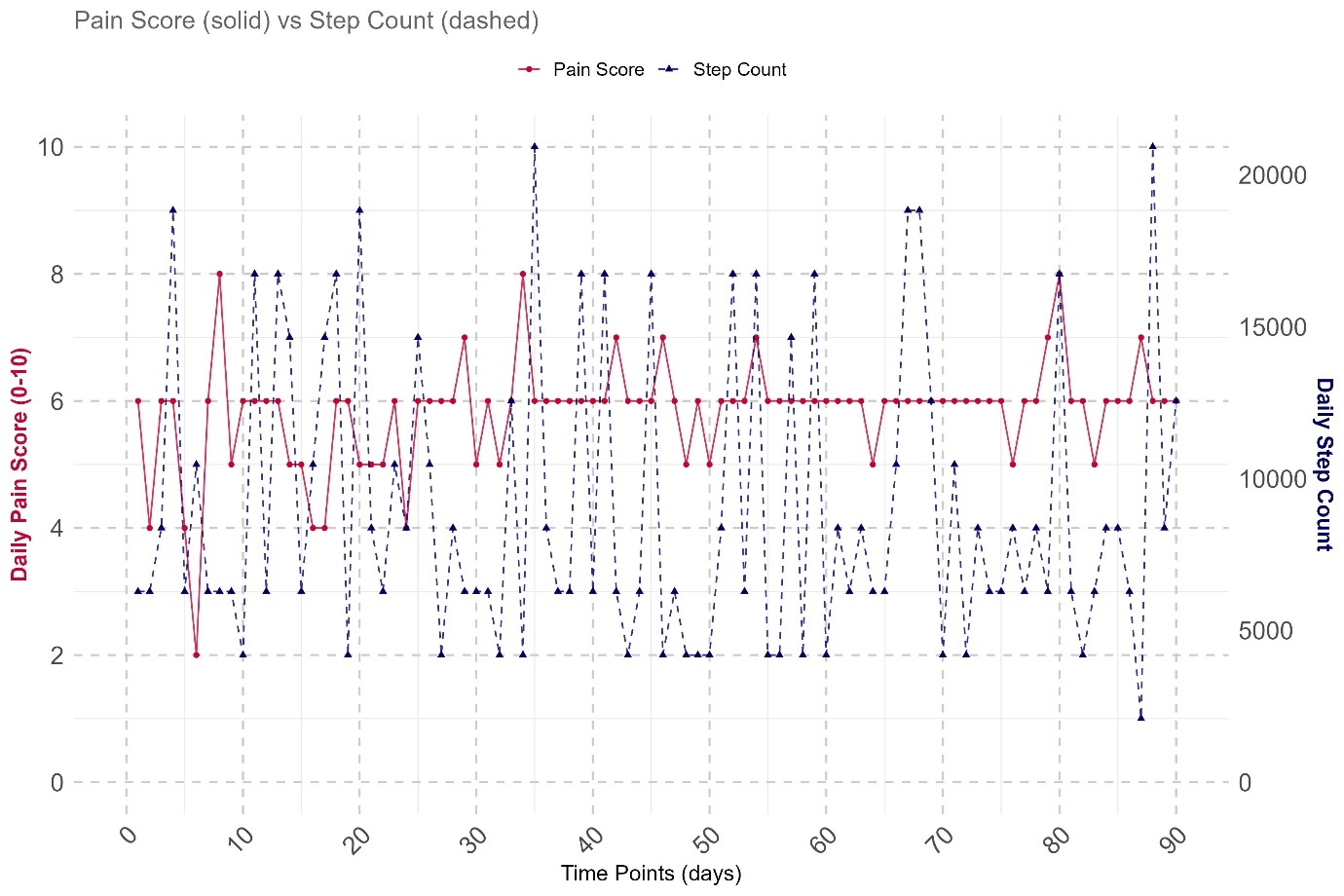
 Figure 2: Daily pain and step count over time for Participant 38.


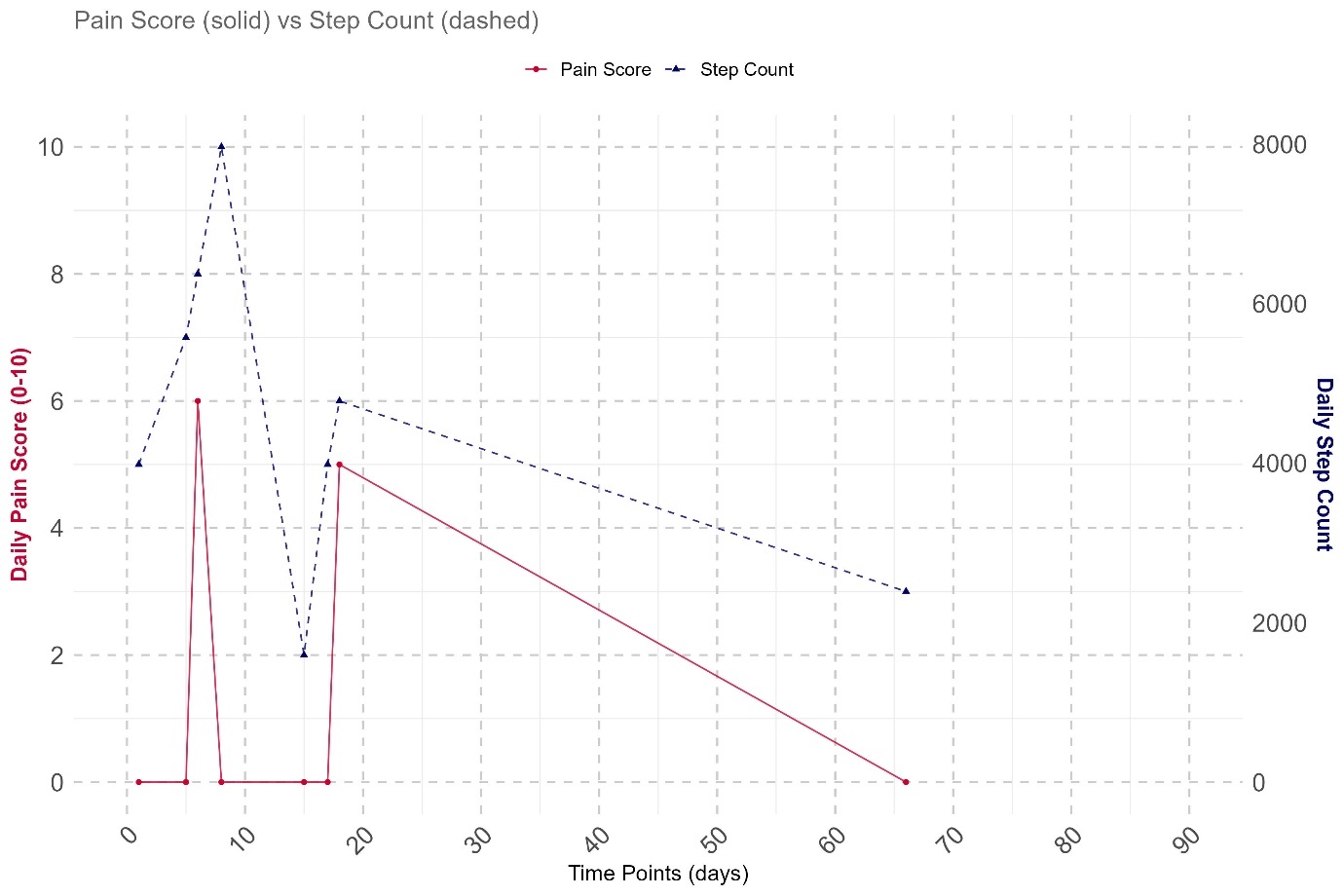


Figure 3: Daily pain and step count over time for Participant 37.


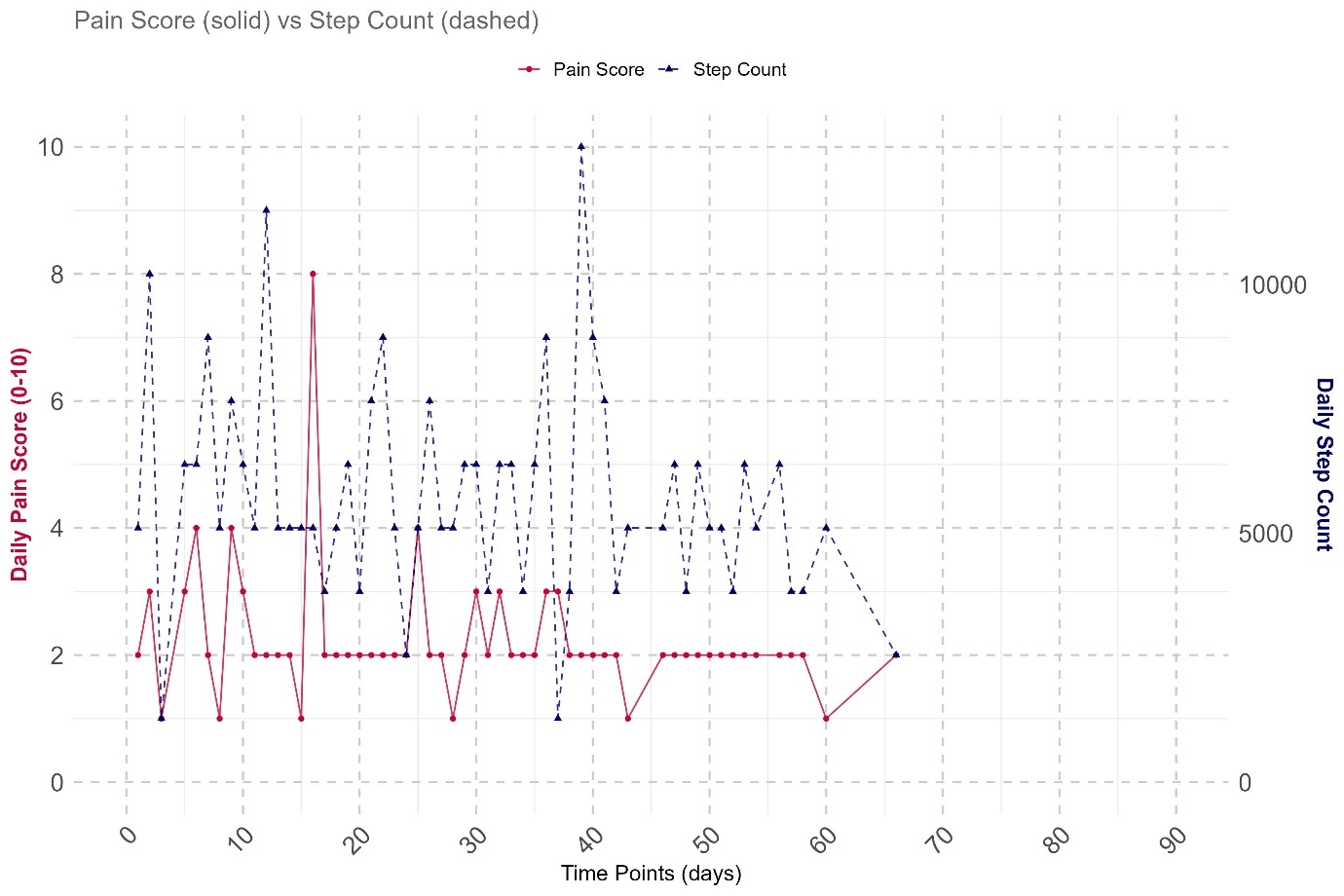
 Figure 4: Daily pain and step count over time for Participant 35.


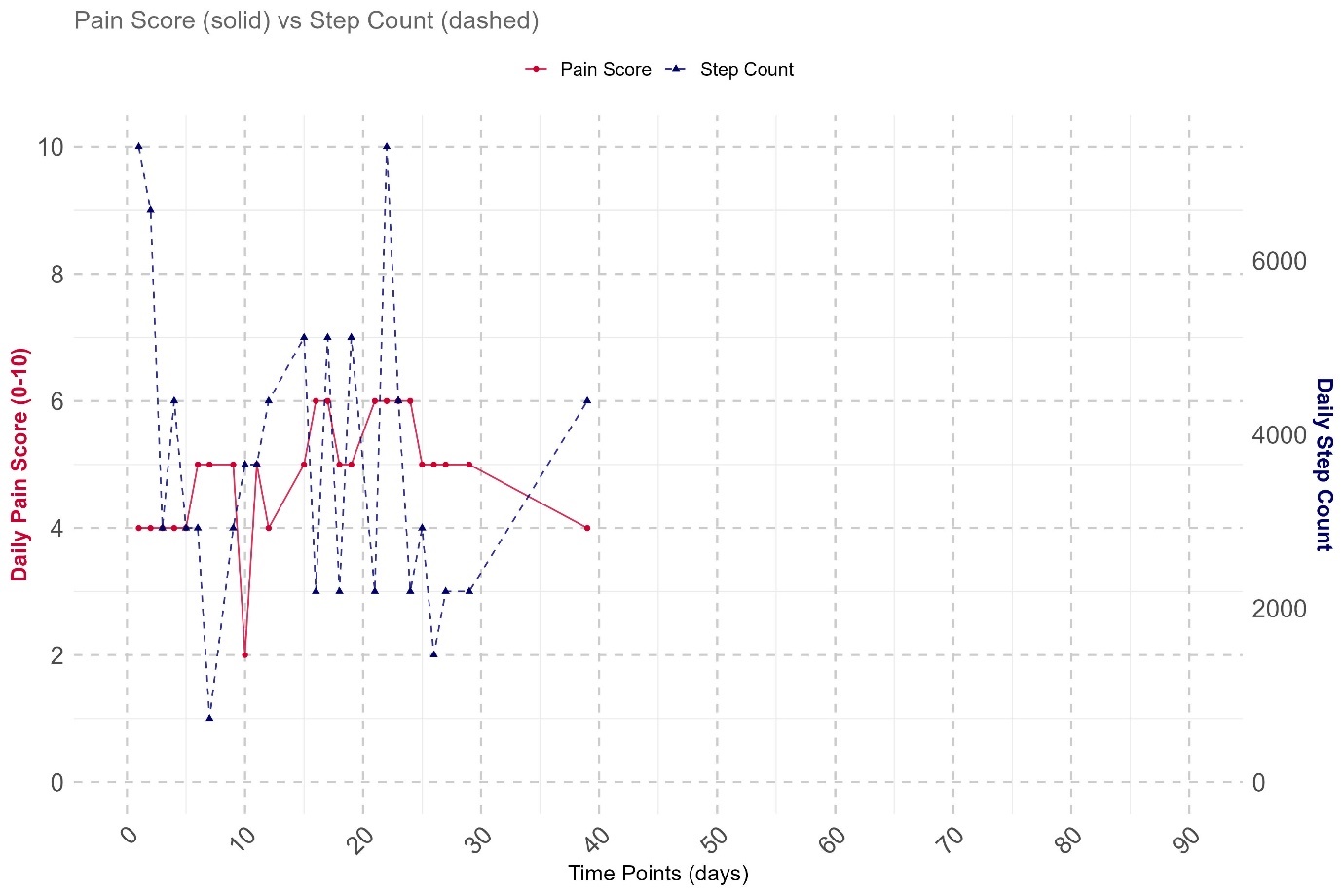


Figure 5: Daily pain and step count over time for Participant 34.


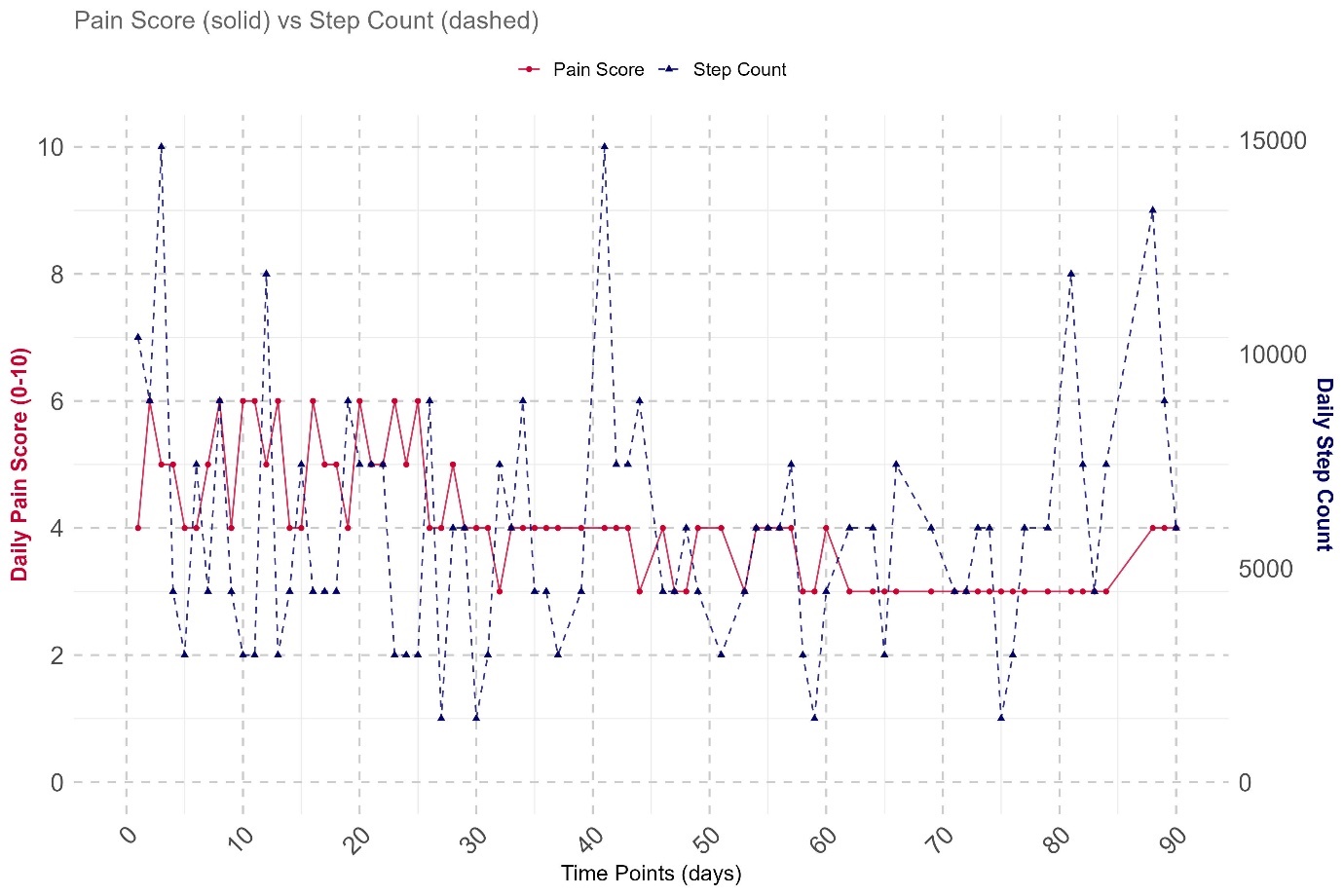


Figure 6: Daily pain and step count over time for Participant 28.


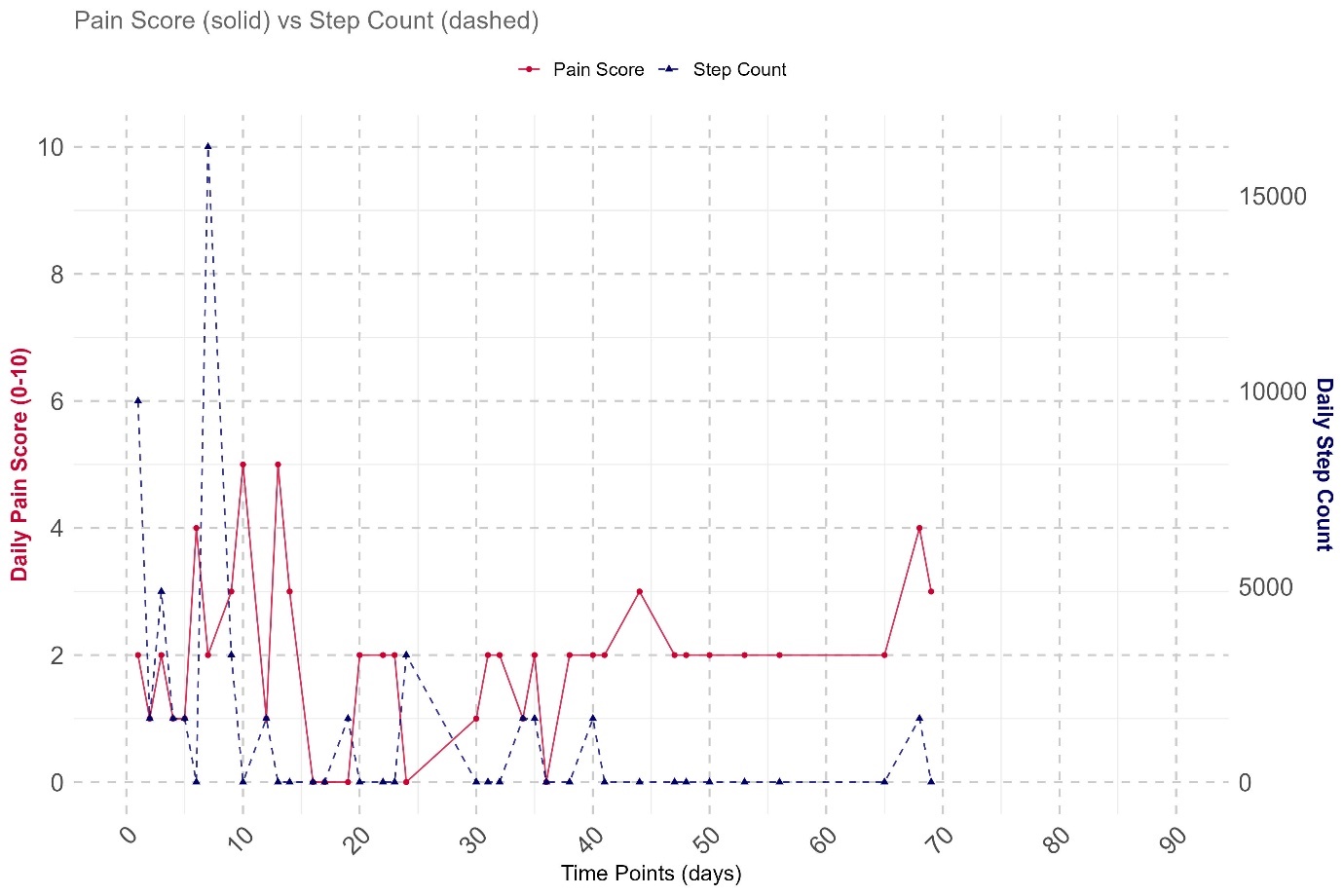
 Figure 7: Daily pain and step count over time for Participant 27.


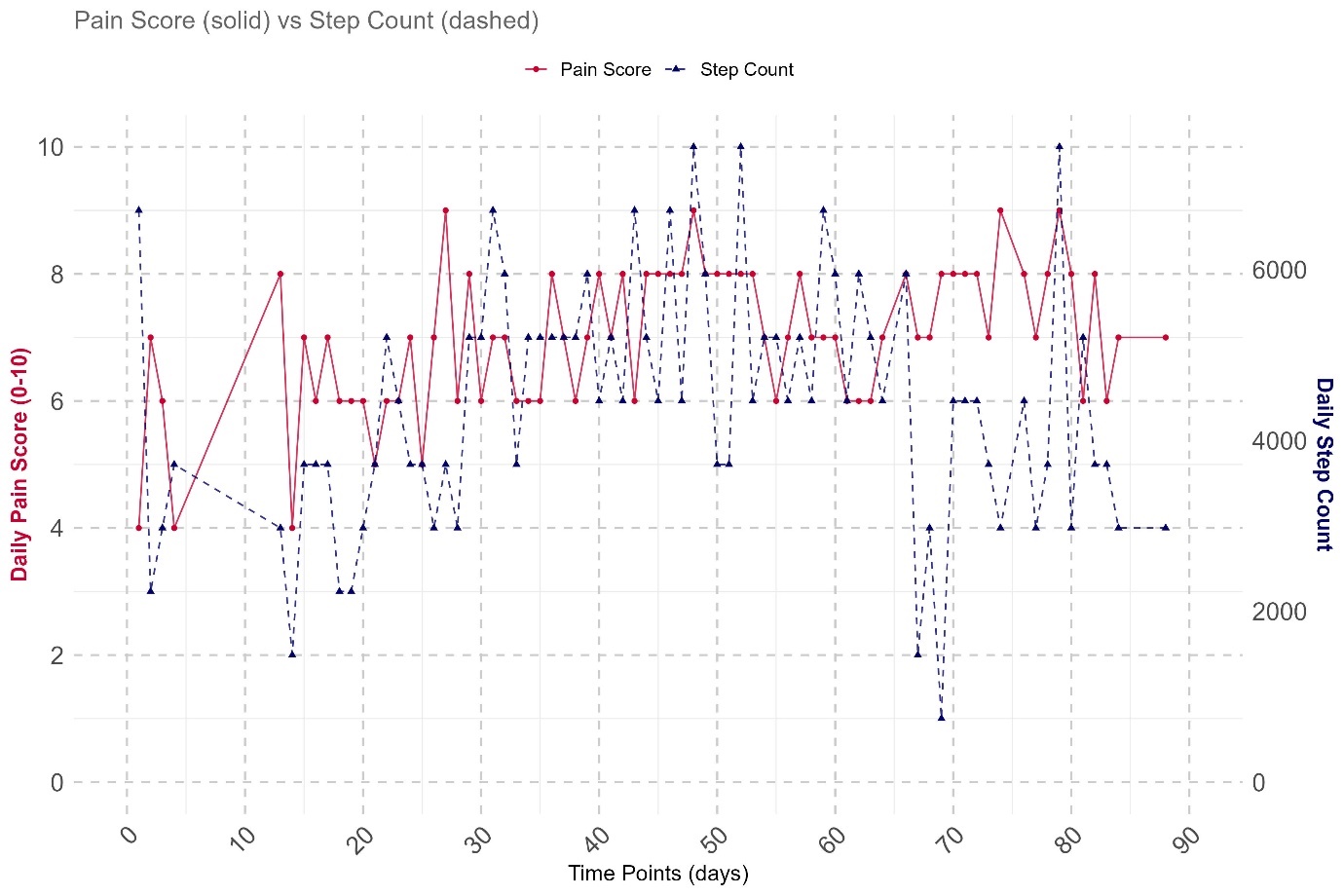
 Figure 8: Daily pain and step count over time for Participant 26.


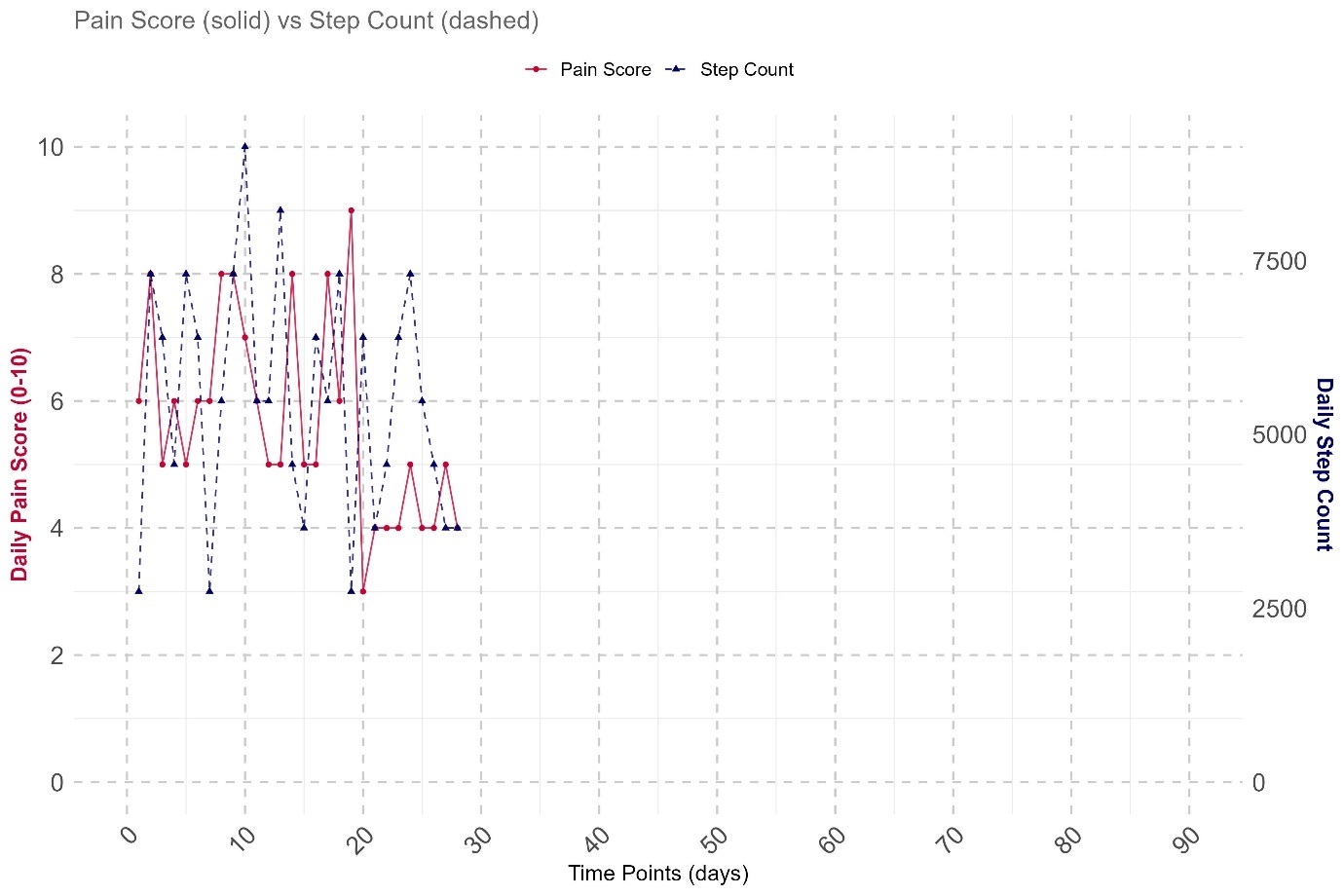


Figure 9: Daily pain and step count over time for Participant 25.


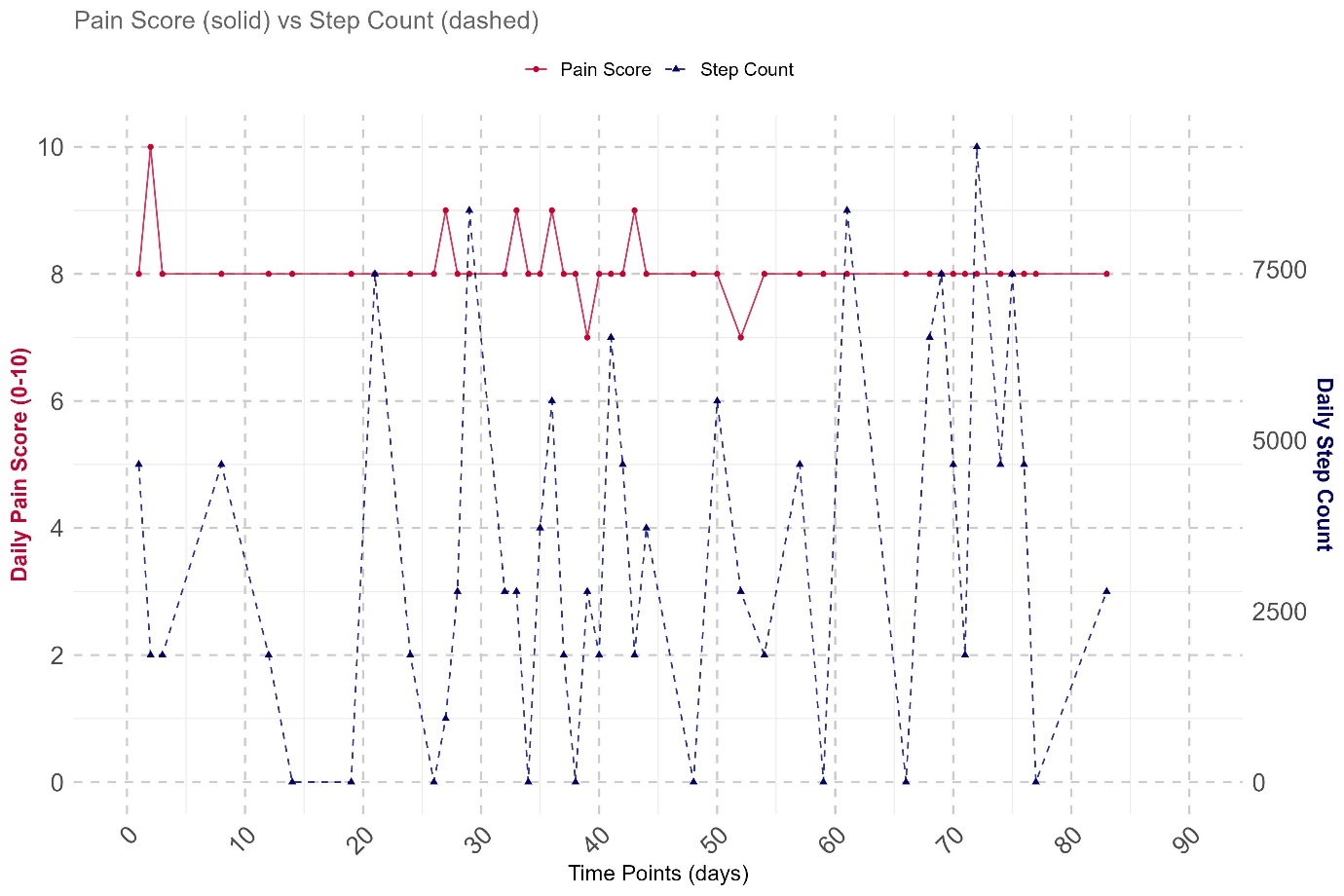
 Figure 10: Daily pain and step count over time for Participant 23.


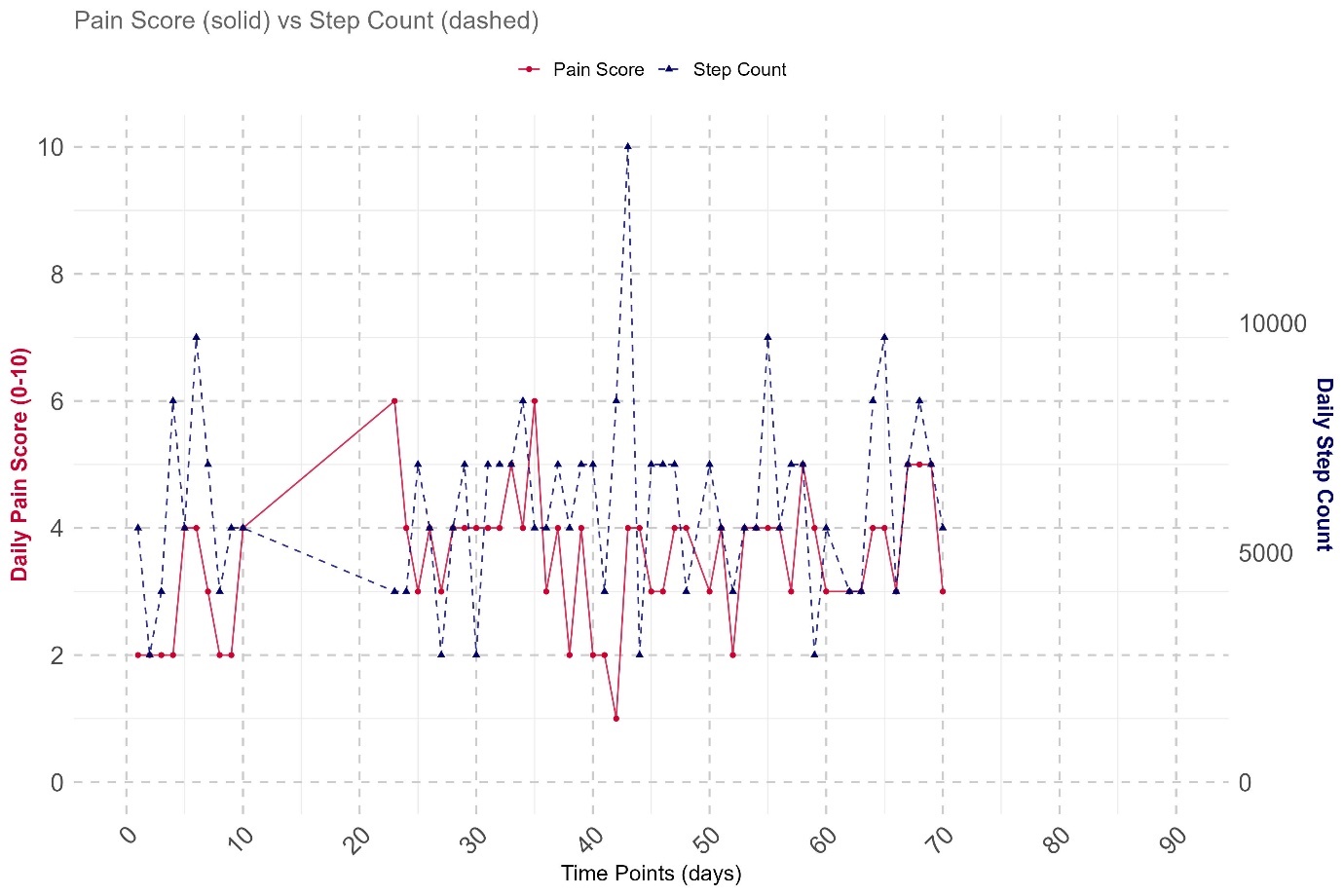


Figure 11: Daily pain and step count over time for Participant 20.


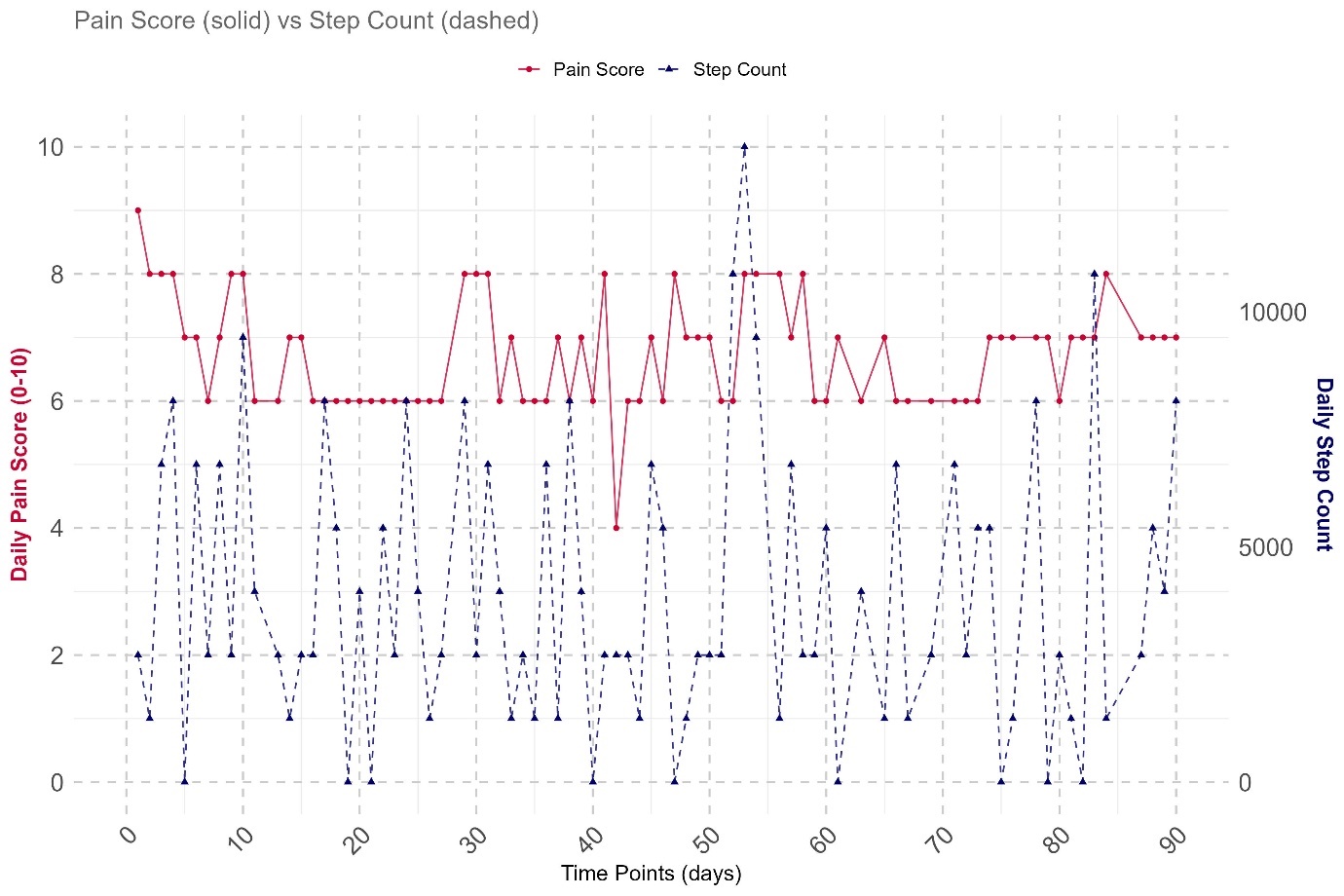
 Figure 12: Daily pain and step count over time for Participant 19.


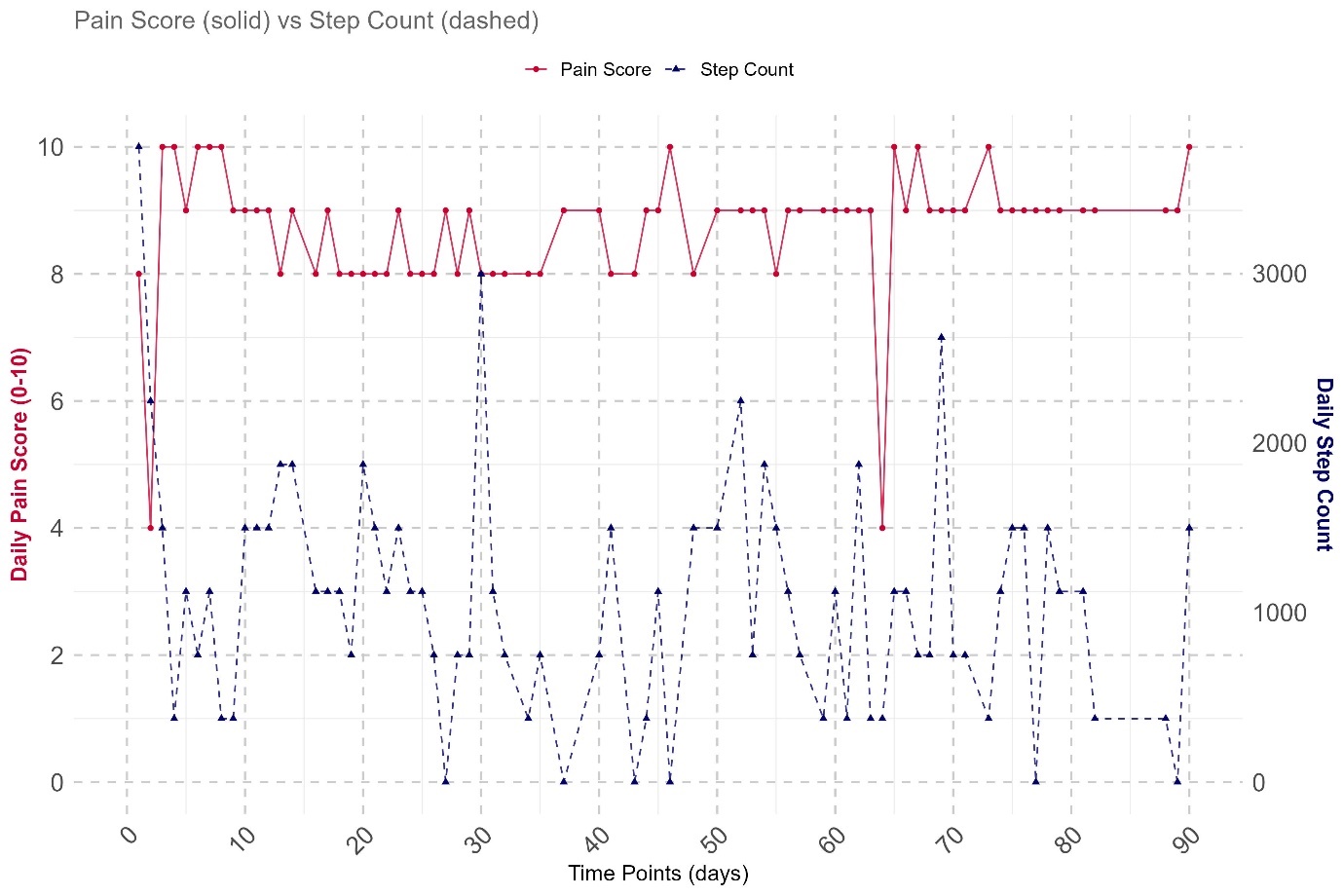


Figure 13: Daily pain and step count over time for Participant 18.


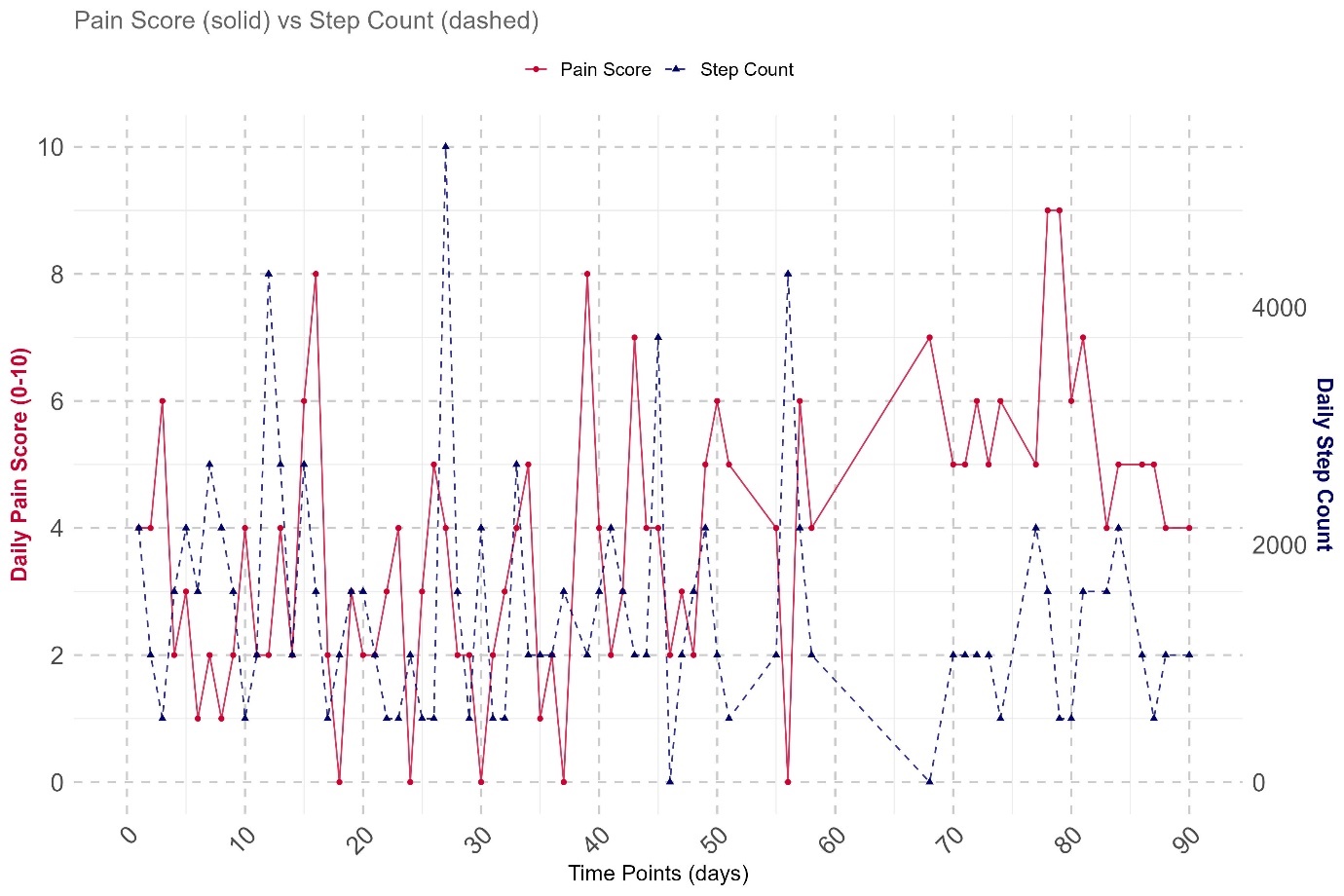
 Figure 14: Daily pain and step count over time for Participant 17.


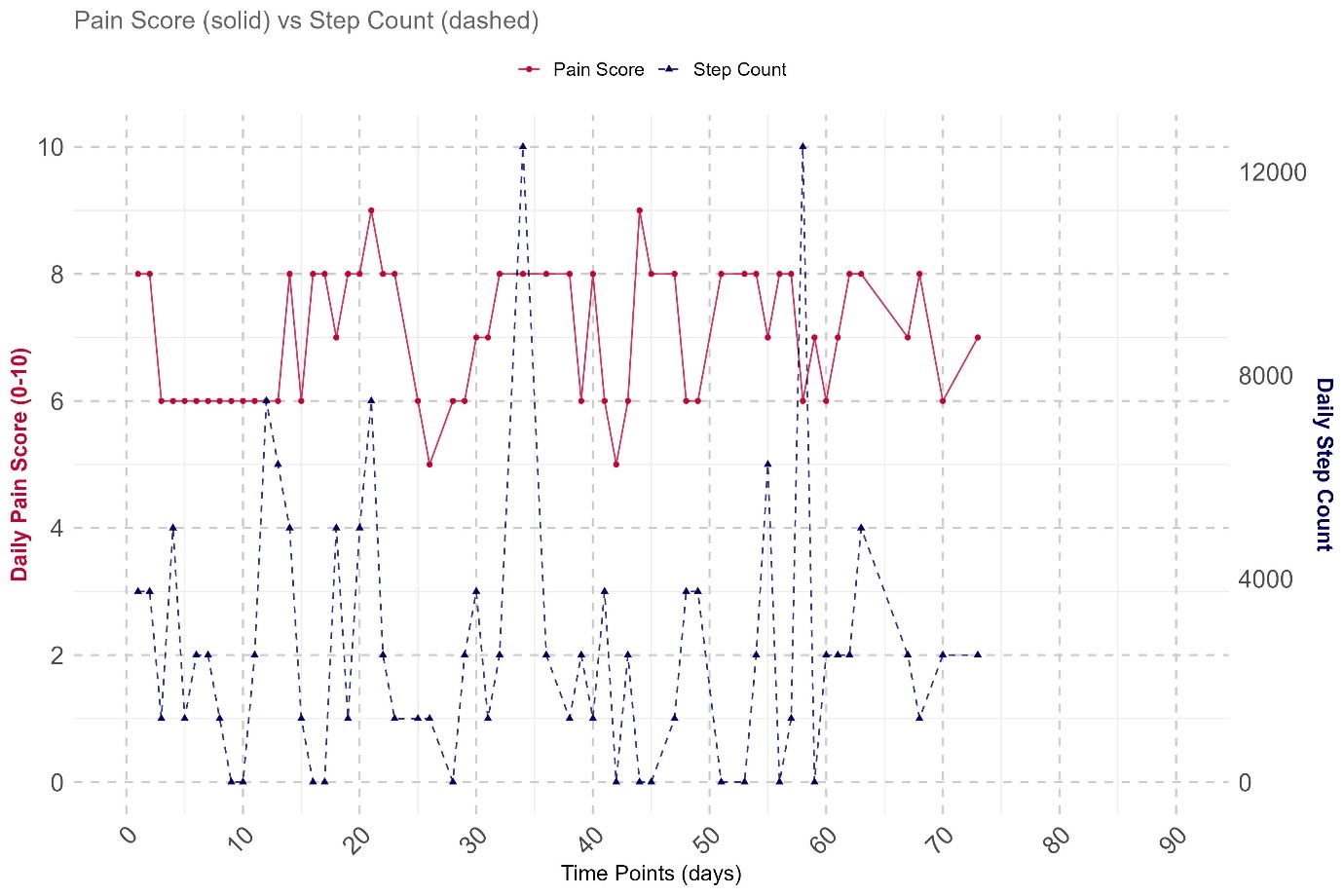


Figure 15: Daily pain and step count over time for Participant 16.


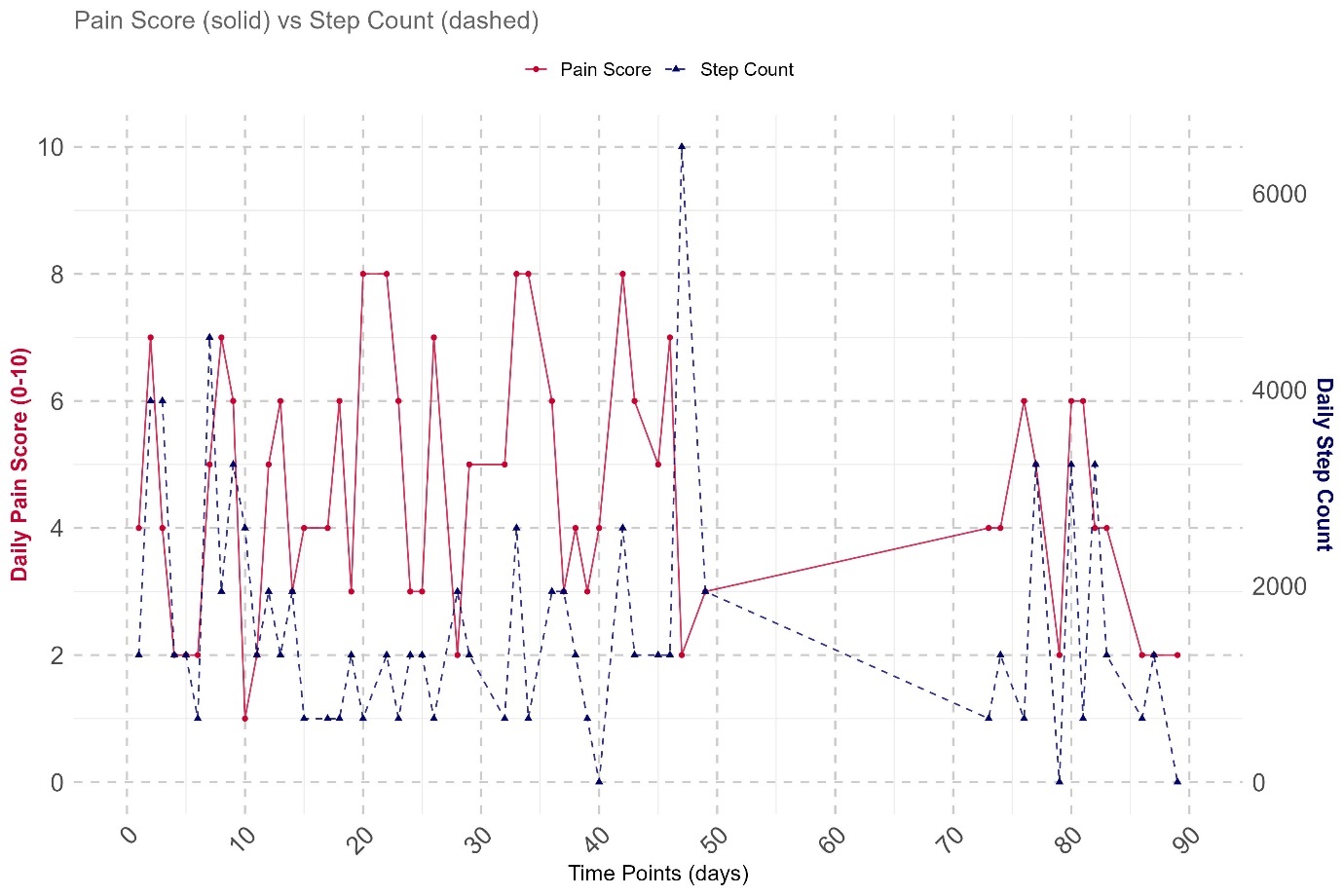
Figure 16: Daily pain and step count over time for Participant 15.


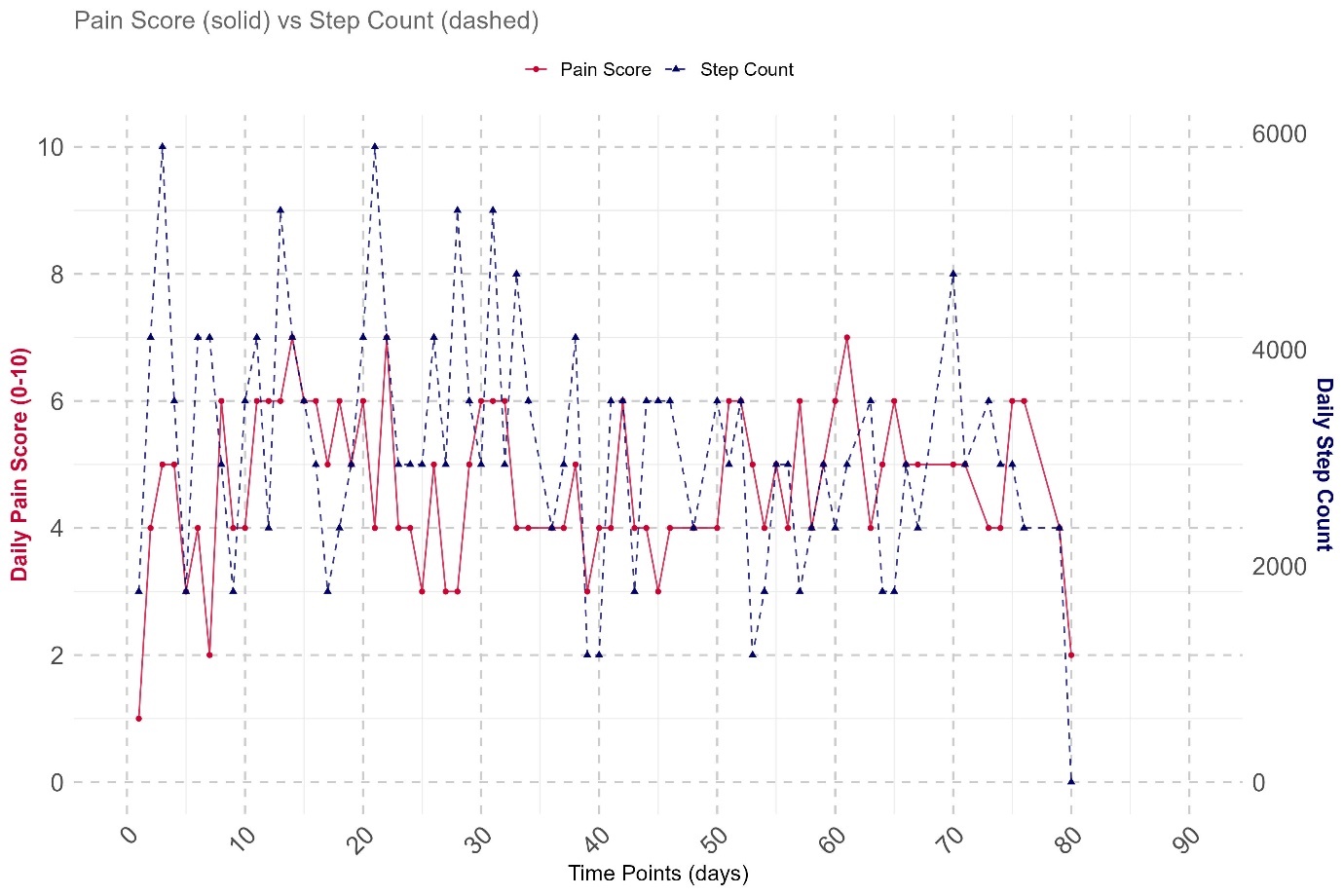


Figure 17: Daily pain and step count over time for Participant 14.


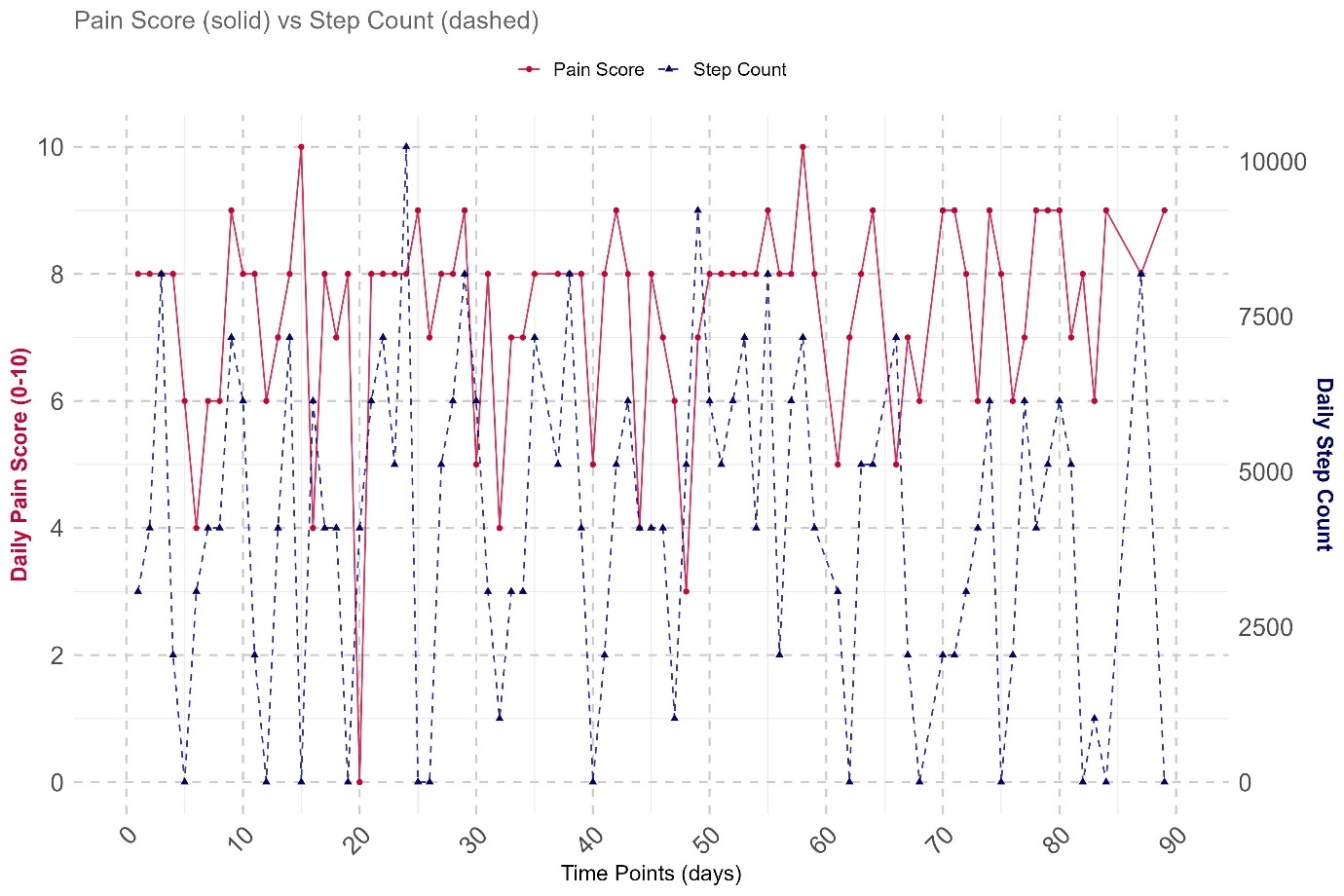
 Figure 18: Daily pain and step count over time for Participant 13.


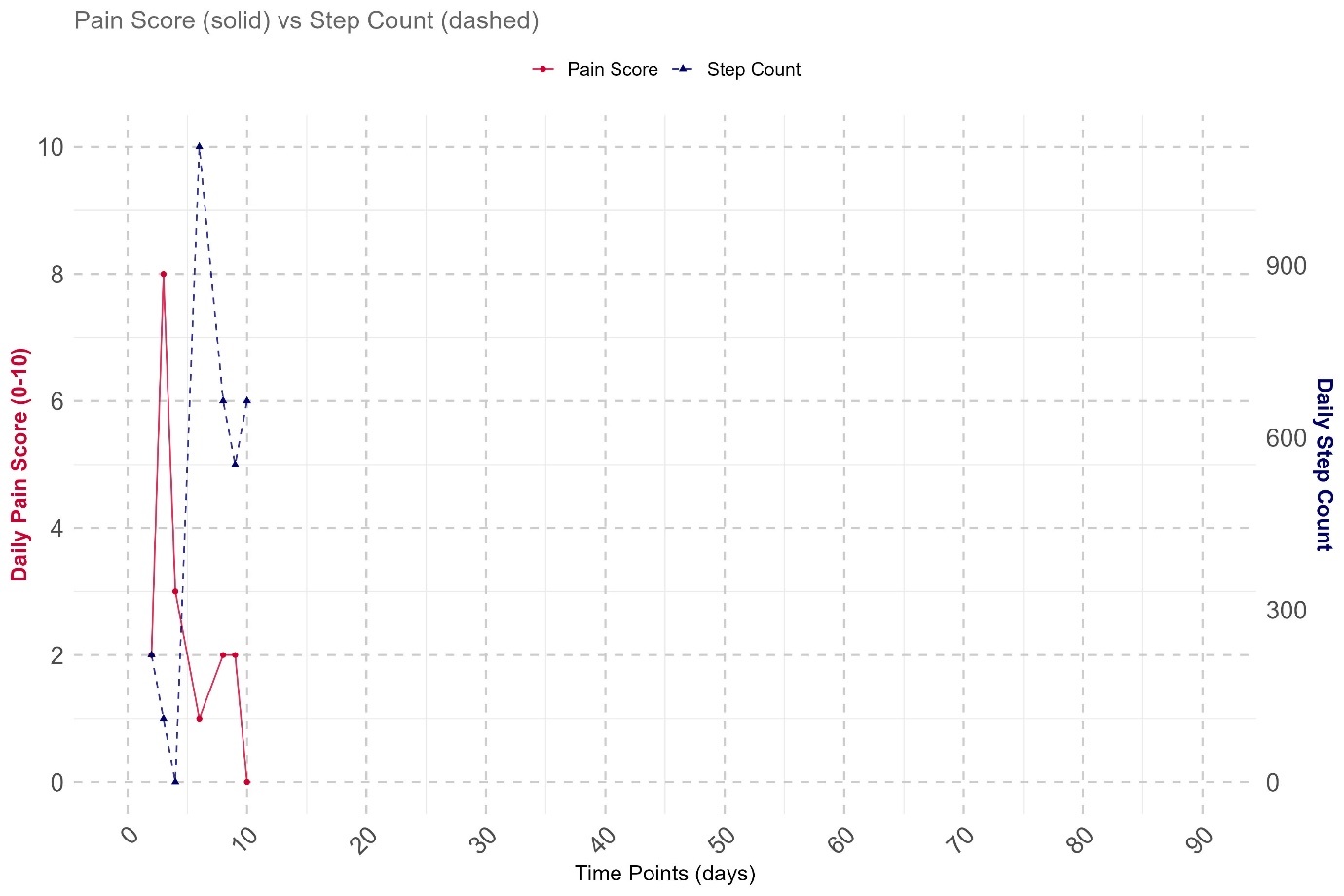


Figure 19: Daily pain and step count over time for Participant 10.


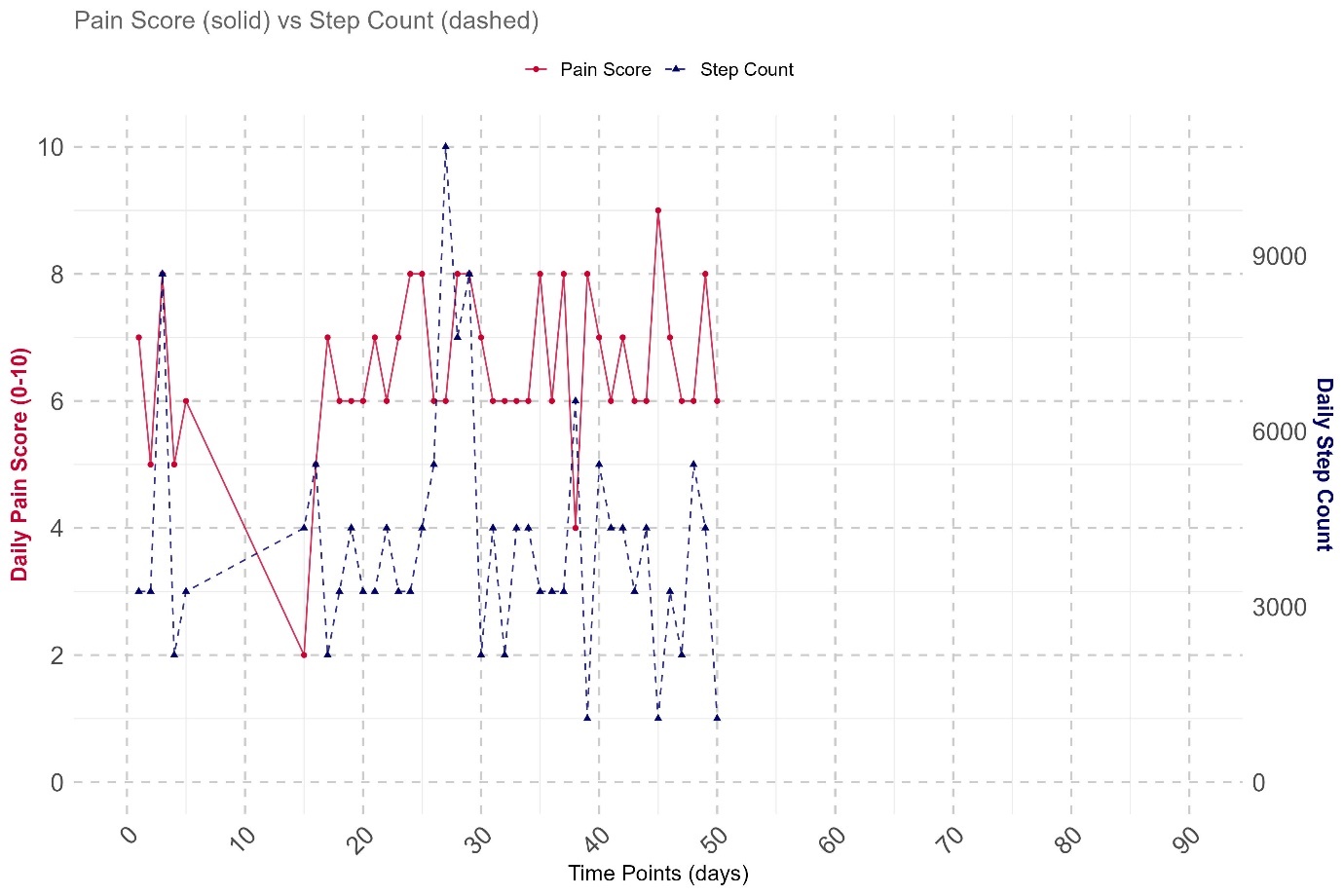
 Figure 20: Daily pain and step count over time for Participant 9.


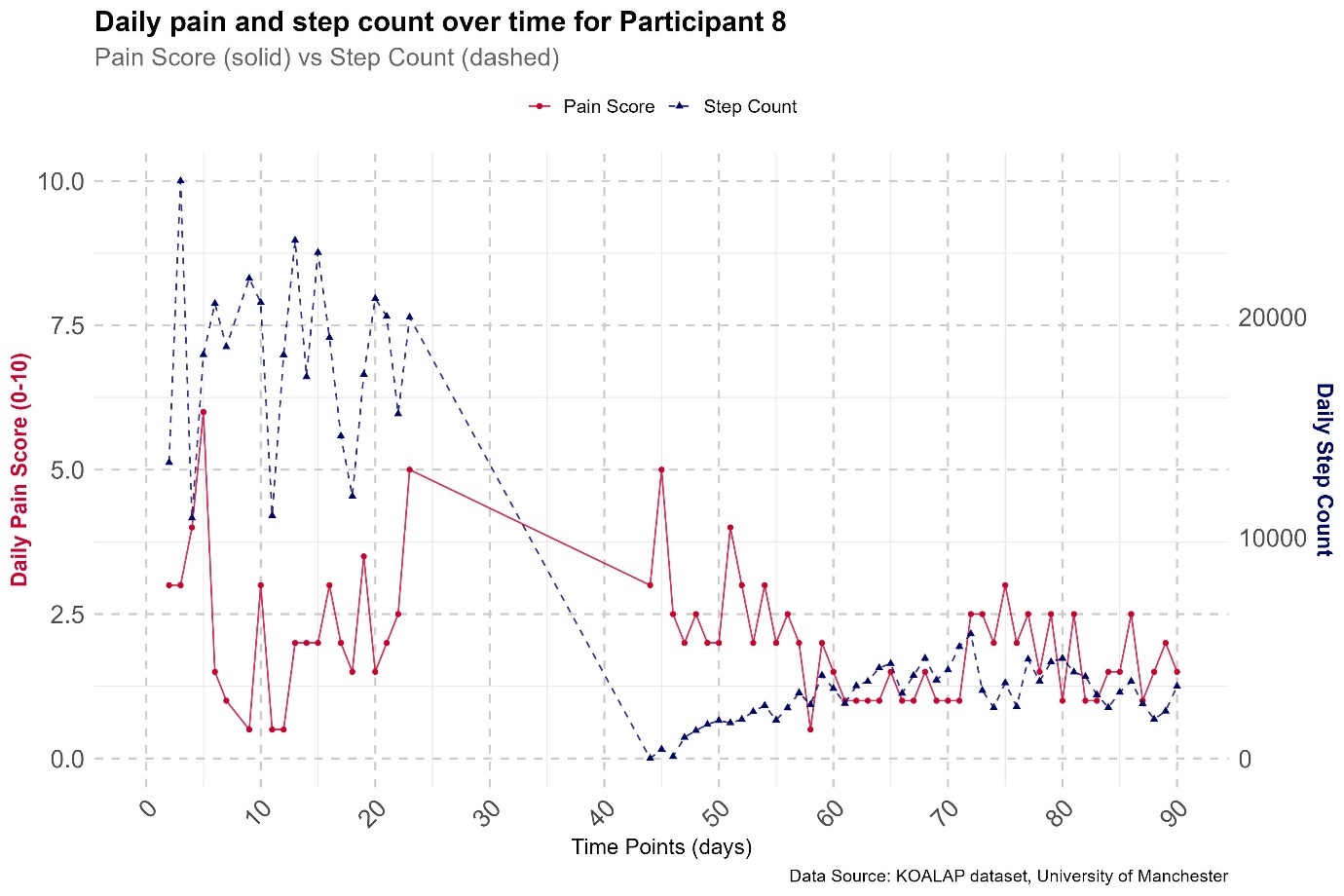


Figure 21: Daily pain and step count over time for Participant 8.


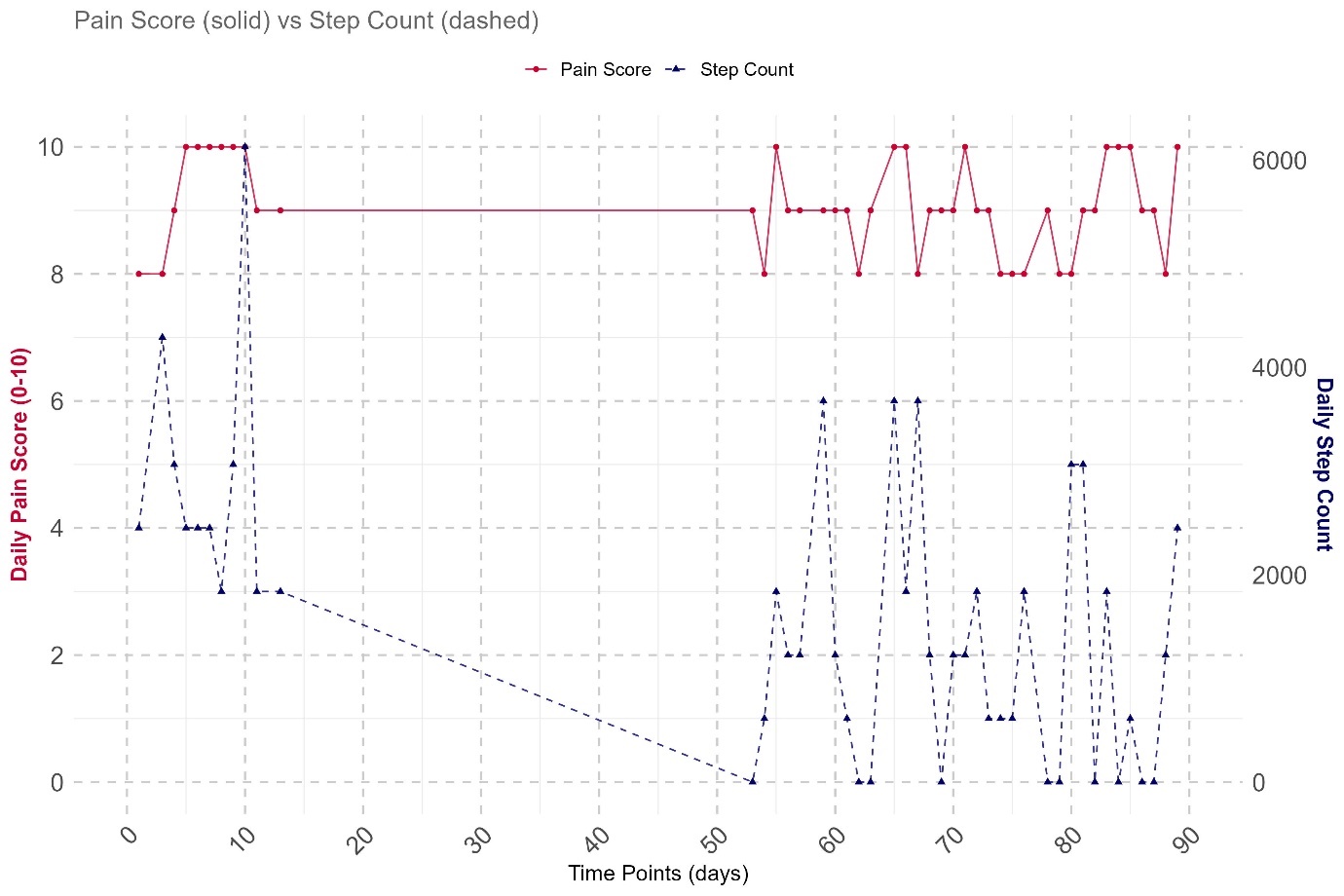
 Figure 22: Daily pain and step count over time for Participant 7.


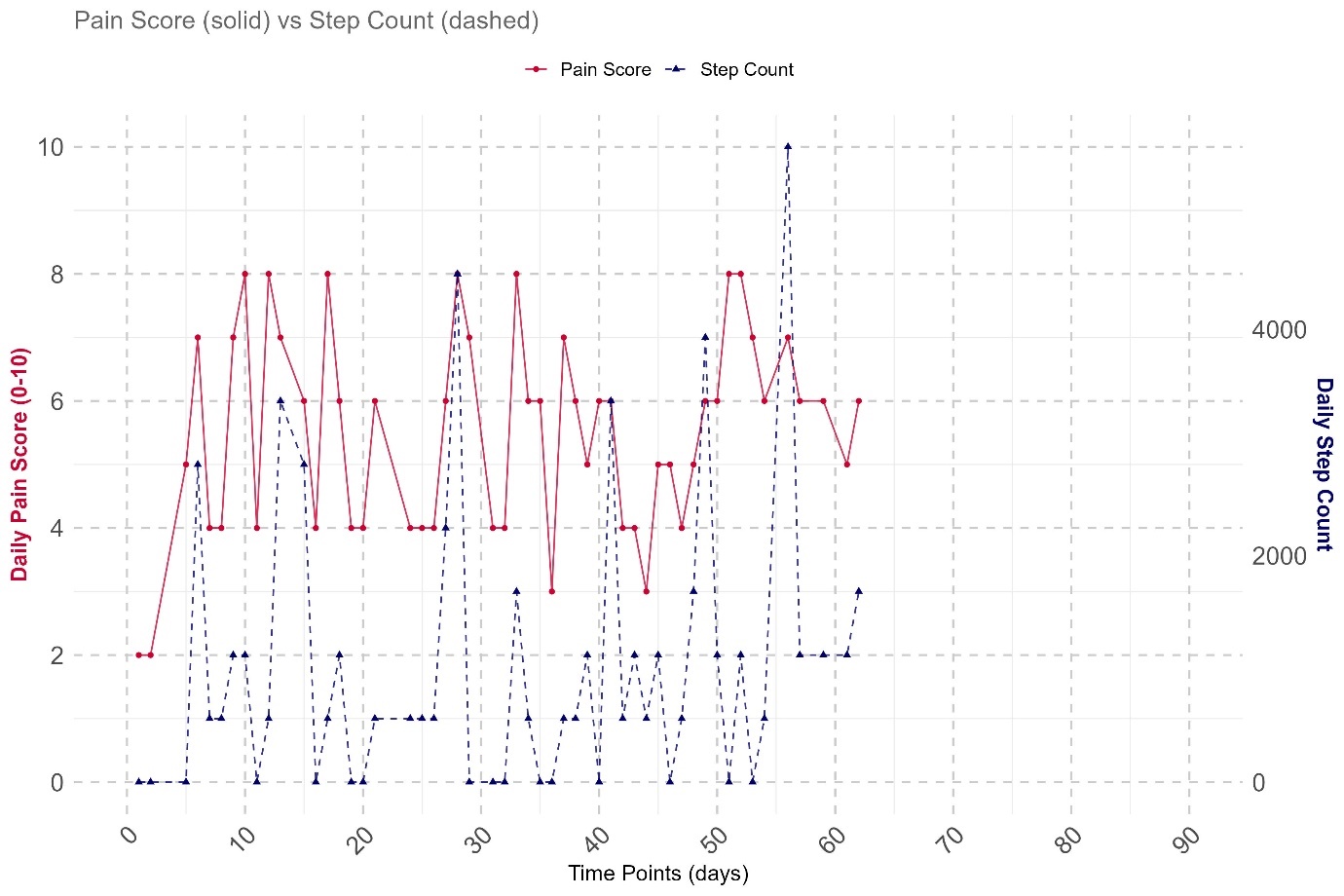


Figure 23: Daily pain and step count over time for Participant 5.


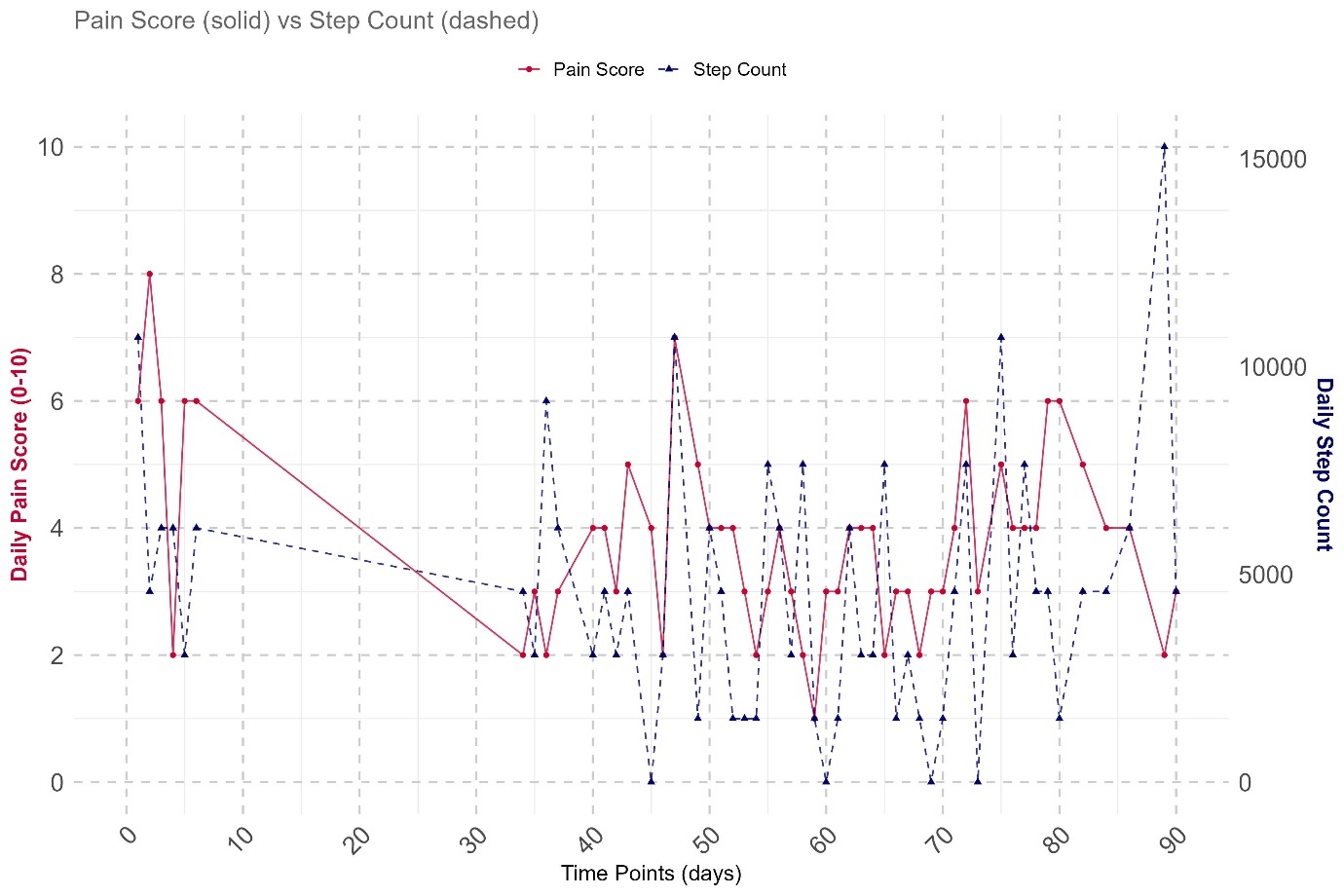
 Figure 24: Daily pain and step count over time for Participant 3.

**Section B:** Association between pain and step count (per 1000 unit increase in step count) or step count and pain (per 1 unit increase in NRS pain) for each participant plus whole population (“Group”) estimate (Model 2 to Model 5)


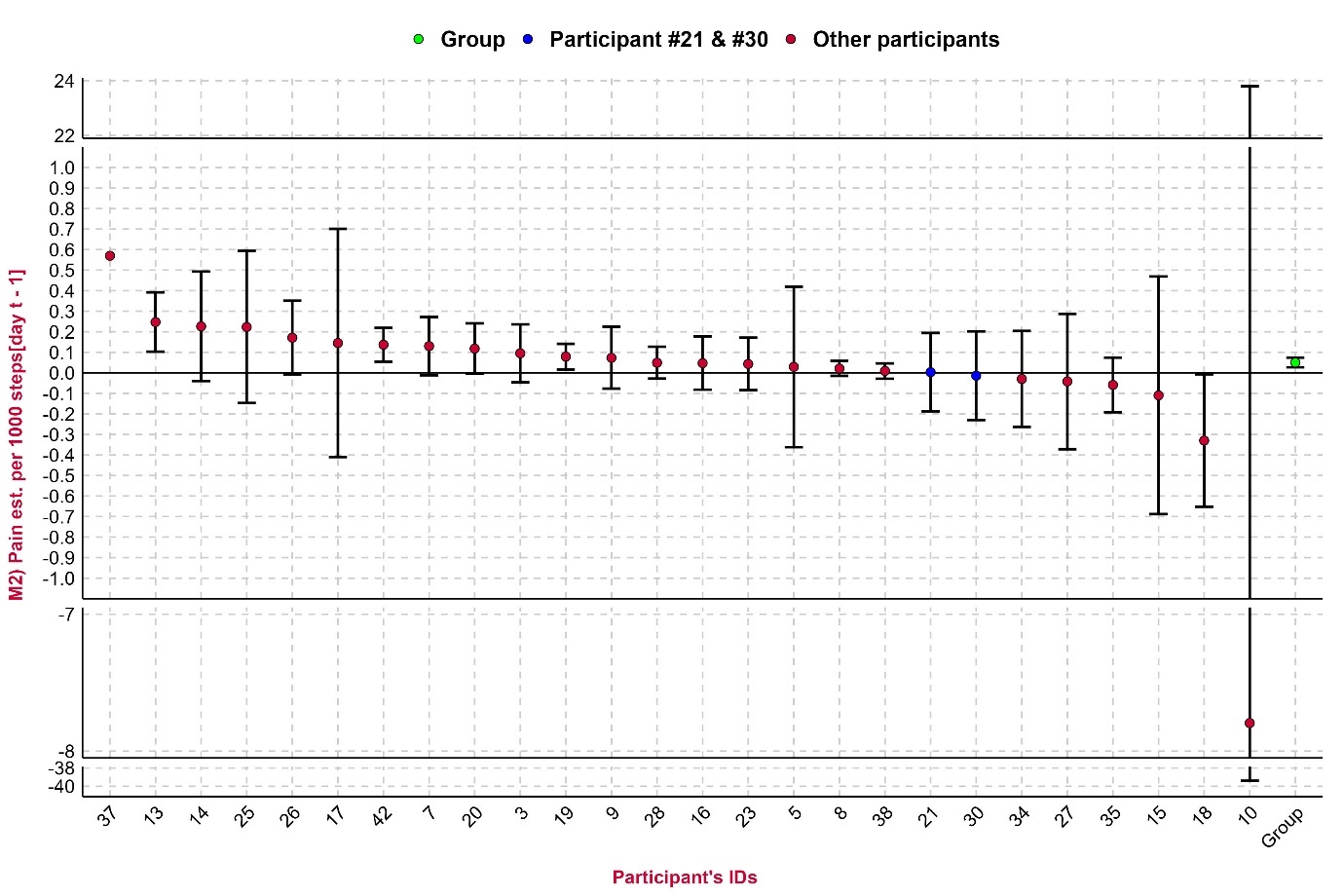


Figure B1: Association between the current day’s pain and prior day’s step count (per 1000 unit increase in step count) for each participant plus whole population (“Group”) estimate (M2).

The preceding M is a short form for Model 2 in Table 2. M2) = the current day’s pain [day t] and prior day’s step count [day t - 1]. est. = estimate. Day t = current day. Each participant is represented within the plot, ranked by their strength of association, with the vertical lines representing confidence intervals (CI). Participants 10 had wide CI and 37 had no CI due to sparse data. The point estimates coloured blue highlight participants 21 and 30 whose pain and step count distributions are shown in both Figure 1 and Figure 2, illustrating where they lie in the population-wide distributions for the analysis. The green point estimate on the right represents the whole population, or the group, estimate which is also reported for Model 2 on Table 2.


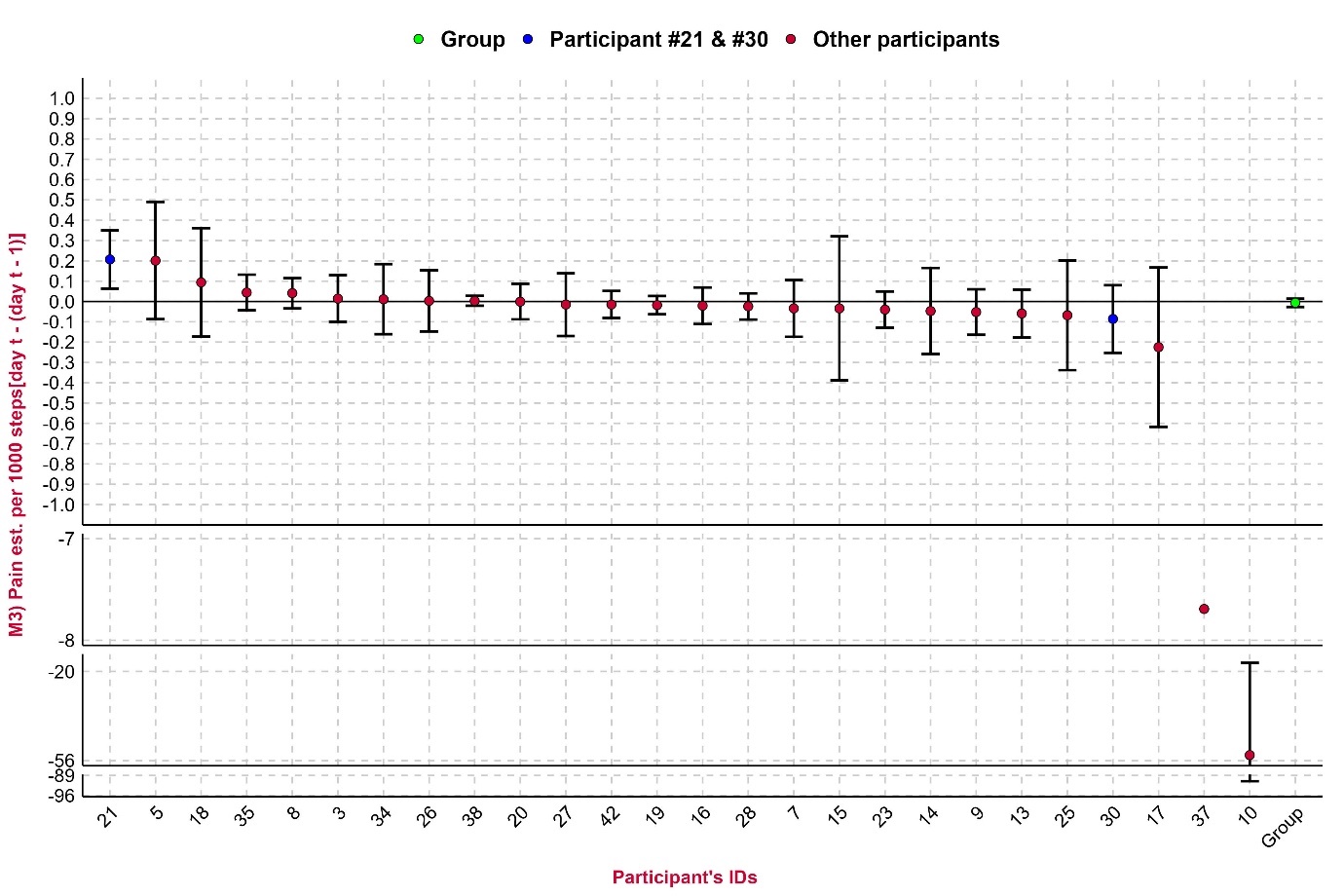


Figure B2: Association between the current day’s pain and change from prior day’s to the current day’s step count (per 1000 unit increase in step count) for each participant plus whole population (“Group”) estimate (M3).

The preceding M is a short form for Model 3 in Table 2. M3) = the current day’s pain [day t]. and change from prior day’s to current day’s step count [(day t) - (day t - 1)]. est. = estimate. Day t = current day. Each participant is represented within the plot, ranked by their strength of association, with the vertical lines representing confidence intervals (CI). Participants 10 had wide CI and 37 had no CI due to sparse data. The point estimates coloured blue highlight participants 21 and 30 whose pain and step count distributions are shown in both Figure 1 and Figure 2, illustrating where they lie in the population-wide distributions for the analysis. The green point estimate on the right represents the whole population, or the group, estimate which is also reported for Model 3 on Table 2.


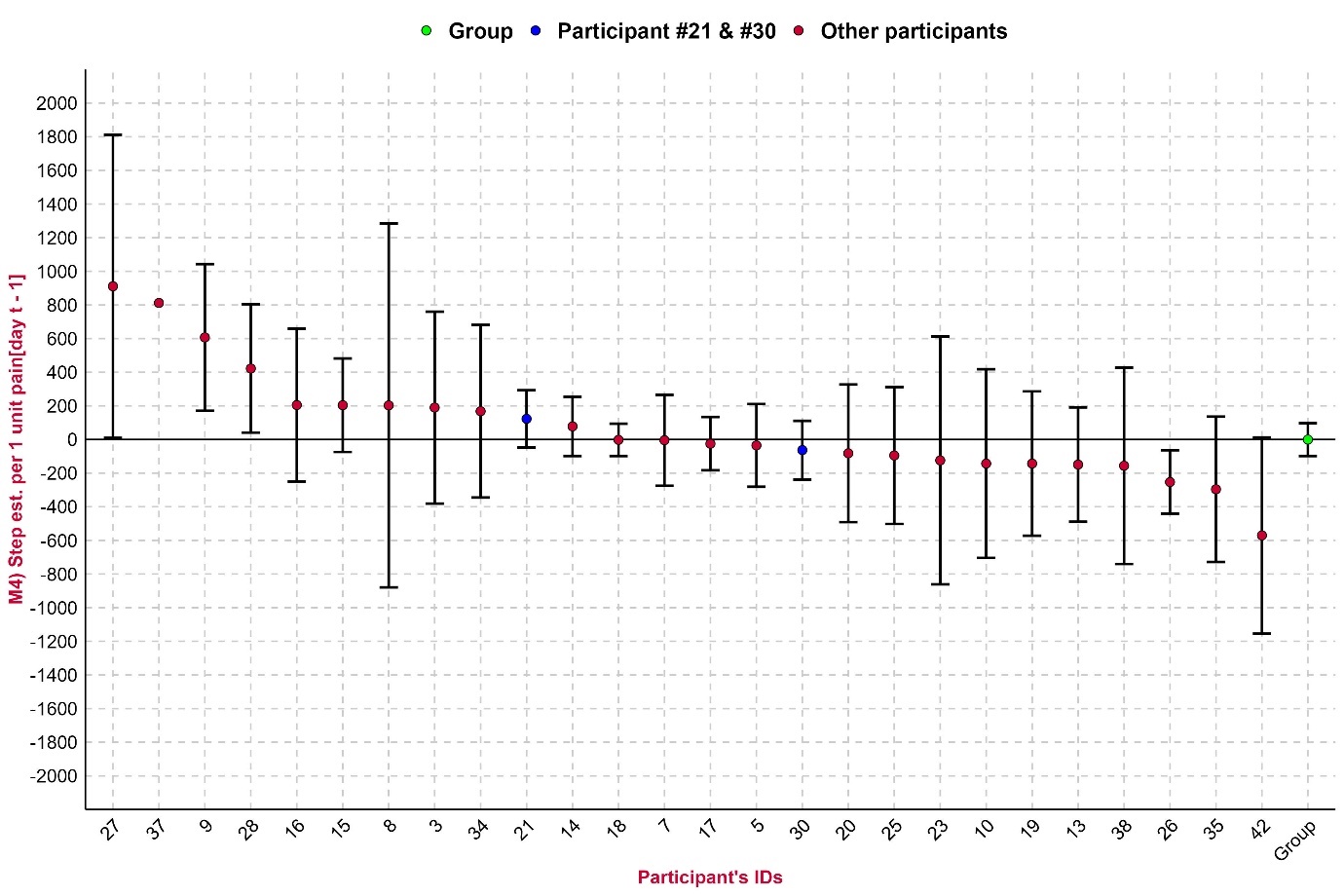


Figure B3: Association between the current day’s step count and prior day’s pain (per 1 unit increase in NRS pain) for each participant plus whole population (“Group”) estimate (M4).

The preceding M is a short form for Model 4 in Table 2. M4) = the current day’s step count [day t] and prior day’s pain [day t -1]. est. = estimate. Day t = current day. Each participant is represented within the plot, ranked by their strength of association, with the vertical lines representing confidence intervals (CI). Participants 10 and 37 had sparse data, thus affecting their results. The point estimates coloured blue highlight participants 21 and 30 whose pain and step count distributions are shown in both Figure 1 and Figure 2, illustrating where they lie in the population-wide distributions for the analysis. The green point estimate on the right represents the whole population, or the group, estimate which is also reported for Model 4 on Table 2.


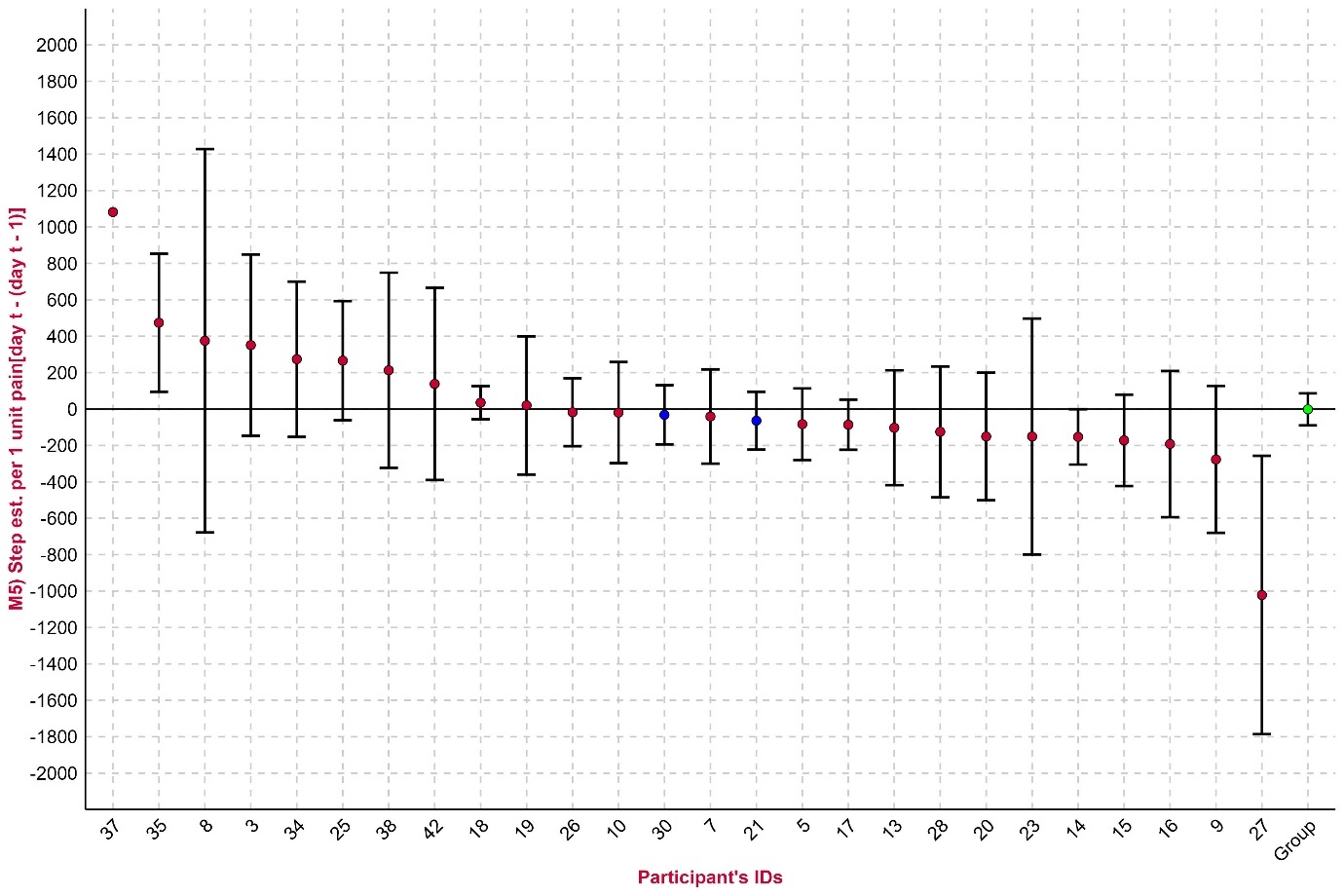


Figure B4: Association between the current day’s step count and change from prior day’s to the current day’s pain (per 1 unit increase in NRS pain) for each participant plus whole population (“Group”) estimate (M5).

The preceding M is a short form for Model 5 in Table 2 M5) = current day’s step count [day t] and change from prior day’s to the current day’s pain [(day t) - (day t - 1)]. est. = estimate. Day t = current day. Each participant is represented within the plot, ranked by their strength of association, with the vertical lines representing confidence intervals (CI). Participants 10 and 37 had sparse data, thus affecting their results. The point estimates coloured blue highlight participants 21 and 30 whose pain and step count distributions are shown in both Figure 1 and Figure 2, illustrating where they lie in the population-wide distributions for the analysis. The green point estimate on the right represents the whole population, or the group, estimate which is also reported for Model 5 on Table 2.
