# Supplementary material for: Exploring the bidirectional temporal association between daily knee pain and physical activity in people with knee osteoarthritis: An exploratory smartwatch study
Source: Osteoarthr Cartil Open. 2026 Jan 31;8(1):100753. doi: 10.1016/j.ocarto.2026.100753 (PMC12907850; doi:10.1016/j.ocarto.2026.100753)
Supplement: Multimedia component 2 [file mmc2.zip › Supplementary_file_2_24Dec2025.html]

Supplementary file to ‘Exploring the bidirectional temporal association between daily knee pain and physical activity in people with knee osteoarthritis: an exploratory smartwatch study.’


Code 

- Show All Code
- Hide All Code

# Supplementary file to ‘Exploring the bidirectional temporal association between daily knee pain and physical activity in people with knee osteoarthritis: an exploratory smartwatch study.’

```
**Author:** Ayobami Olanrewaju 
**Code Reviewer:** Emma Pritchard  
**Last updated:** Dec 24, 2025
```

Click *Show* to view the code.

### Libraries used

- lubridate
- ggplot2
- dplyr
- tidyverse
- Matrix
- lme4
- broom
- ggbreak
- MASS
- lmerTest
- patchwork
- broom.mixed
- readxl
- effectsize
- performance
- knitr

### About the Data

At the time of publication, the data is not yet available but is
expected to become available in the future upon request. Please contact
the corresponding author for more information

### Set-up - functions (data prep, modelling & plotting)

```
# Note: All inner_join results were checked to ensure correct behavior and that relevant observations were retained.

# 1: Function to create pain and step lag by a day
   # -i.e., replace current day's pain and step count with yesterday's
lagging_function <- function(df, column_name)
{
  # Basic checks: stop function if more than one row of data per user per day
  dup_check <- df %>%
  dplyr::count(user_id, timestamp.Day) %>%
  dplyr::filter(n > 1)
  if (nrow(dup_check) > 0) {
  stop("Multiple rows per user per day detected in lagging_function()")
  }
  
  # Once it passes the check, it creates a dataframe with value for lagged pain and lagged steps.
  laggged_df <- df %>%
  arrange(user_id, timestamp.Day) %>%  # Sort by user_id and date
  group_by(user_id) %>%  #group by user_id to lag step and pain per user_id
  mutate(lagged_pain = ifelse(
    timestamp.Day - lag(timestamp.Day) == 1,  # Check if the previous row is the previous day
    lag(df[[column_name]]),  # Assign previous day's pain value to current day
    NA  # Assign NA if date is not consecutive
  ), lagged_steps = ifelse(
    timestamp.Day - lag(timestamp.Day) == 1,  # Check if the previous row is the previous day
    lag(steps),  # Assign previous day's step value to current day
    NA  # # Assign NA if date is not consecutive
  )) %>%
  ungroup() 
  
  return(laggged_df)
}

# 2: Function to create change from prior day to current day pain and step 
   #- i.e., compute difference between current and prior day's pain and step. 
change_function <- function(df, column_name)
{
  # Basic checks: stop function if more than one row of data per user per day
  dup_check <- df %>%
  dplyr::count(user_id, timestamp.Day) %>%
  dplyr::filter(n > 1)
  if (nrow(dup_check) > 0) {
  stop("Multiple rows per user per day detected in change_function()")
  }
  
  # Once it passes the check, it creates a dataframe with value for change pain and change steps.
  change_df <- df %>%
  arrange(user_id, timestamp.Day) %>%  # Sort by user_id and date
  group_by(user_id) %>%  # Group by user_id to lag step and pain per user_id
  mutate(change_in_pain = ifelse(
    timestamp.Day - lag(timestamp.Day) == 1,  # Check if the previous row is the previous day
    df[[column_name]] - lag(df[[column_name]], 1),  # Compute change pain value
    NA  # Assign NA if date is not consecutive
  ), change_in_steps = ifelse(
    timestamp.Day - lag(timestamp.Day) == 1,  # Check if the previous row is the previous day
    steps - lag(steps, 1),  # Compute change step value 
    NA  # Assign NA if date is not consecutive
  )) %>%
  ungroup() 
  
  return(change_df)
}

# 3: Count number of records contributed by each participants
count_function <- function(df, col_name)
  {

  count_results <-  df %>%
  group_by(user_id) %>%
  summarise (
    records_no = sum(!is.na(.data[[col_name]]))
    )
  return(count_results)
}

# 4: Get summary stats (median value) for pain and step count for all participant
get_users_summary_stats_function <- function(df, col_name_1, col_name_2)
  {

  users_summary_stats_results <-  df %>%
  group_by(user_id) %>%
  summarise (
    #Get median value for pain for all users
    median_pain = round(median(.data[[col_name_1]])),
  
   #Get median value  for steps for all users
    median_step = round(median(.data[[col_name_2]]))
  )
   
  # Return both medians
  return(users_summary_stats_results)
}


# 5: Association Across the population - Generalised Linear Mixed model fitting method
across_id_lmer_function <- function(df,
                          col_name,
                          predictor_names,
                          ran_effect_name,
                          ci_method = c("Wald", "profile"),
                          steps_multiplier = 1000) {

  ci_method <- match.arg(ci_method)

  # Allow user to pass a single string or a character vector
  predictor_names <- as.character(predictor_names)

  # Basic checks: stops function if needed columns are missing
  needed <- c(col_name, predictor_names, ran_effect_name)
  missing_cols <- setdiff(needed, names(df))
  if (length(missing_cols) > 0) {
    stop("Missing columns in df: ", paste(missing_cols, collapse = ", "))
  }

  # Build formula: `y` ~ `x1` + `x2` + ... + (1 | `group`)
  fixed_part <- paste(sprintf("`%s`", predictor_names), collapse = " + ")
  model_formula <- stats::as.formula(
    sprintf("`%s` ~ %s + (1 | `%s`)", col_name, fixed_part, ran_effect_name)
  )

  # Fit model
  mod <- lmerTest::lmer(model_formula, data = df, REML = TRUE)

  # Tidy fixed effects (Satterthwaite df/p-values)
  out <- broom.mixed::tidy(mod, effects = "fixed", ddf.method = "Satterthwaite")

  # CIs for fixed effects
  ci <- suppressMessages(confint(mod, method = ci_method, parm = "beta_"))
  ci_df <- data.frame(
    term = rownames(ci),
    lower_ci = ci[, 1],
    upper_ci = ci[, 2],
    row.names = NULL,
    check.names = FALSE
  )
  out <- dplyr::left_join(out, ci_df, by = "term")

  # Save unscaled estimate and 95%CI
  out <- dplyr::mutate(
    out,
    estimate_raw = estimate,
    lower_ci_raw = lower_ci,
    upper_ci_raw = upper_ci
  )

  # ---- Steps scaling: apply to any predictor whose name contains "steps" ----
  steps_preds <- predictor_names[grepl("steps", predictor_names, ignore.case = TRUE)]
  if (length(steps_preds) > 0) {
    # tidy() term names are usually like "steps" or "`steps`" depending on tooling
    # so match either bare or backticked
    steps_term_pattern <- paste0("^(`)?(", paste(steps_preds, collapse = "|"), ")(`)?$")
    is_steps_term <- grepl(steps_term_pattern, out$term)

    out <- dplyr::mutate(
      out,
      estimate = dplyr::if_else(is_steps_term, estimate * steps_multiplier, estimate),
      lower_ci = dplyr::if_else(is_steps_term, lower_ci * steps_multiplier, lower_ci),
      upper_ci = dplyr::if_else(is_steps_term, upper_ci * steps_multiplier, upper_ci)
    )
  }

  # ---- Standardized beta effect sizes for numeric predictors ----
  out$effect_std_beta <- NA_real_
  out$effect_std_beta_lo <- NA_real_
  out$effect_std_beta_hi <- NA_real_

  y <- df[[col_name]]
  sy <- stats::sd(y, na.rm = TRUE)

  for (i in seq_len(nrow(out))) {
    term_i <- out$term[i]
    if (term_i == "(Intercept)") next

    # strip backticks so we can look up in df
    term_clean <- gsub("`", "", term_i)

    # Skip factor-expanded terms like factorB, poly(...), interactions, etc.
    if (!term_clean %in% names(df)) next

    x <- df[[term_clean]]
    if (!is.numeric(x) || !is.numeric(y) || is.na(sy) || sy == 0) next

    sx <- stats::sd(x, na.rm = TRUE)
    if (is.na(sx) || sx == 0) next

    out$effect_std_beta[i]    <- out$estimate_raw[i] * (sx / sy)
    out$effect_std_beta_lo[i] <- out$lower_ci_raw[i] * (sx / sy)
    out$effect_std_beta_hi[i] <- out$upper_ci_raw[i] * (sx / sy)
  }

  list(
    lmer_model = mod,
    lmer_model_results = out
  )
}

# 5a: Function to rounding up models 4 and 5 whose result is in step count and others to 2 digits
round_for_model_4_and_5 <- function(x, model_name) {
  if (model_name %in% c("Model_4", "Model_5")) {
    if (abs(x) >= 1) {
      formatC(round(x, 0), format = "f", digits = 0)
    } else {
      formatC(round(x, 1), format = "f", digits = 1)
    }
  } else {
    formatC(round(x, 2), format = "f", digits = 2)
  }
}

##5aa: Function to rounding up models 4 and 5 whose result is in step count and others to 3 digits
round_for_model_4_and_5_b <- function(x, model_name) {
  if (model_name %in% c("Model_4", "Model_5")) {
    if (abs(x) >= 1) {
      formatC(round(x, 0), format = "f", digits = 0)
    } else {
      formatC(round(x, 1), format = "f", digits = 1)
    }
  } else {
    formatC(round(x, 3), format = "f", digits = 3)
  }
}

# 5b: Function to round up effect size to 2dp or max of 4dp
smart_round_effect <- function(x, max_dp = 4) {
  if (is.na(x)) return(NA_real_)
  if (x == 0) return(0)

  for (dp in 2:max_dp) {
    r <- round(x, dp)
    if (r != 0) {
      return(formatC(r, format = "f", digits = dp))
    }
  }

  # Max of 4dp rounding (very tiny values)
   formatC(x, format = "f", digits = max_dp)
}

# 6: Association with the population - ordinary least square model fitting method
within_users_ols_function <- function(df, col_name, predictor_name, steps_multiplier = 1000)
{
  # Fit linear model and extract regression results for each group
ols_model_results <- df %>%
  group_by(user_id) %>%
  group_modify( ~{
    
    # Fit the linear model for each group
    ols_model <- lm(as.formula(paste(col_name, "~", predictor_name)), data = .)
    
    # Use broom to tidy the model output
    tidy_model <- tidy(ols_model)
    
    # Extract confidence intervals
    conf_intervals <- confint(ols_model)
    
    
    
    # Scaling steps" so that it is per 1000 steps instead of per 1 step
  is_steps <- grepl("steps", predictor_name, ignore.case = TRUE)
  if (is_steps) {
    tidy_model <- tidy_model %>%
    dplyr::mutate(
      estimate = ifelse(term == predictor_name, estimate * steps_multiplier, estimate),
      # Add confidence intervals to the tidy output
      lower_ci = ifelse(term == predictor_name, conf_intervals[term, 1] * steps_multiplier, conf_intervals[term, 1]),
      upper_ci = ifelse(term == predictor_name, conf_intervals[term, 2] * steps_multiplier, conf_intervals[term, 2])
    )
} else {
  # Add CI to the tidy output without scaling if predictor does not contain steps
  tidy_model <- tidy_model %>%
    dplyr::mutate(
      lower_ci = conf_intervals[term, 1],
      upper_ci = conf_intervals[term, 2]
    )
}
    
    # Return the results
    return(tidy_model)
  })
return(ols_model_results) 
}

# 6a:Convert all model results into type double
convert_to_dbl <- function(df) {
  df %>%
  dplyr::mutate(
    dplyr::across(
      matches("^M[1-5]_Est(_low_ci|_up_ci)?$"),
      as.numeric
    )
  )
}

# 7a: Make ordered plots without y-axis break for Model 1 to model 5 results
make_ordered_ci_plot <- function(
  df,
  est_col,
  low_ci_col,
  up_ci_col,
  y_scale_name,
  y_limits,
  y_breaks,
  y_label,
  x_label = "Participant's IDs",
  highlight_ids = c("21", "30"),
  group_id = "Group",
  legend_position = "none"
) {
  # Basic checks: stops function if needed columns are missing
  stopifnot(is.data.frame(df))
  needed <- c("user_id", est_col, low_ci_col, up_ci_col)
  missing <- setdiff(needed, names(df))
  if (length(missing) > 0) {
    stop("Missing columns in df: ", paste(missing, collapse = ", "))
  }

  # Prepare data and ordering (keep user_id as character)
  df2 <- df %>%
    dplyr::mutate(user_id = as.character(user_id)) %>%
    dplyr::arrange(user_id == group_id, dplyr::desc(.data[[est_col]])) %>%
    dplyr::mutate(
      color_group = dplyr::case_when(
        user_id %in% highlight_ids ~ "highlight",
        user_id == group_id ~ "different",
        TRUE ~ "normal"
      )
    )

  x_order <- unique(df2$user_id)

  # Plot
  p <- ggplot2::ggplot(df2, ggplot2::aes(x = user_id, y = .data[[est_col]])) +
    ggplot2::scale_x_discrete(limits = x_order) +
    ggplot2::geom_errorbar(
      ggplot2::aes(
        ymin = .data[[low_ci_col]],
        ymax = .data[[up_ci_col]]
      ),
      width = 0.4,
      size = 0.8
    ) +
    ggplot2::scale_y_continuous(
      name = y_scale_name,
      limits = y_limits,
      breaks = y_breaks
    ) +
    ggplot2::geom_hline(yintercept = 0, color = "black", linetype = "solid") +
    ggplot2::geom_point(
      ggplot2::aes(fill = color_group),
      shape = 21,
      size = 2.5,
      stroke = 0.4
    ) +
    ggplot2::labs(y = y_label, x = x_label) +
    ggplot2::scale_fill_manual(
      name = NULL,
      values = c("highlight" = "blue", "normal" = "#CC0033", "different" = "green"),
      labels = c(
        "highlight" = paste0("Participant #", paste(highlight_ids, collapse = " & #")),
        "normal" = "Other participants",
        "different" = group_id
      )
    ) +
    ggplot2::scale_color_manual(
      values = c("highlight" = "blue", "normal" = "#CC0033", "different" = "green")
    ) +
    ggplot2::theme_minimal() +
    ggplot2::theme(
      plot.title = ggplot2::element_text(face = "bold", size = 14),
      plot.subtitle = ggplot2::element_text(size = 10, color = "gray40"),
      axis.title.y.left = ggplot2::element_text(color = "#CC0033", face = "bold", size = 12),
      axis.title.x = ggplot2::element_text(color = "#CC0033", face = "bold", size = 12),
      axis.text.x = ggplot2::element_text(angle = 45, hjust = 1, color = "black", size = 12),
      axis.text.y.left = ggplot2::element_text(color = "black", size = 10),
      panel.grid.major = ggplot2::element_line(linetype = "dashed", color = "gray80"),
      axis.line.x.bottom = ggplot2::element_line(color = "black"),
      axis.line.y.left = ggplot2::element_line(color = "black"),
      axis.text.y.right = ggplot2::element_blank(),
      panel.grid.minor = ggplot2::element_blank(),
      legend.position = legend_position,
      legend.title = ggplot2::element_blank()
    )

  return(p)
}

# 7b: Function to add y-axis breaks to a plot
add_y_breaks <- function(p, scale_y_breaks) {
  if (is.null(scale_y_breaks) || length(scale_y_breaks) == 0) return(p)

  for (b in scale_y_breaks) {
    p <- p + ggbreak::scale_y_break(b$range, scales = b$scales)
  }
  p
}

# 7c:  Make ordered plots with y-axis break for Model 1 to model 5 results
make_ggbreak_ci_plot <- function(
  df,
  est,
  low_ci,
  up_ci,
  y_axis_name = NULL,
  y_limits = NULL,
  y_breaks = waiver(),
  scale_y_breaks = list(),
  x_label = "Participant's IDs",
  y_label = NULL,
  highlight_ids = c("21", "30"),
  group_id = "Group",
  legend_position = "top"
) {
  # checks
  stopifnot(is.data.frame(df))
  needed <- c("user_id", est, low_ci, up_ci)
  missing <- setdiff(needed, names(df))
  if (length(missing) > 0) stop("Missing columns: ", paste(missing, collapse = ", "))

  # order rows + color group (keep user_id as character)
  df2 <- df %>%
    dplyr::mutate(user_id = as.character(user_id)) %>%
    dplyr::arrange(user_id == group_id, dplyr::desc(.data[[est]])) %>%
    dplyr::mutate(
      color_group = dplyr::case_when(
        user_id %in% highlight_ids ~ "highlight",
        user_id == group_id ~ "different",
        TRUE ~ "normal"
      )
    )

  x_order <- unique(df2$user_id)

  # Base plot
  p <- ggplot2::ggplot(df2, ggplot2::aes(x = user_id, y = .data[[est]])) +
    ggplot2::scale_x_discrete(limits = x_order) +
    ggplot2::geom_errorbar(
      ggplot2::aes(ymin = .data[[low_ci]], ymax = .data[[up_ci]]),
      width = 0.4, size = 0.8
    ) +
    ggplot2::geom_hline(yintercept = 0, color = "black", linetype = "solid") +
    ggplot2::geom_point(ggplot2::aes(fill = color_group), shape = 21, size = 3, stroke = 0.4) +
    ggplot2::scale_fill_manual(
      name = NULL,
      values = c("highlight" = "blue", "normal" = "#CC0033", "different" = "green"),
      labels = c(
        "highlight" = paste0("Participant #", paste(highlight_ids, collapse = " & #")),
        "normal" = "Other participants",
        "different" = group_id
      )
    ) +
    ggplot2::scale_color_manual(
      name = "Color",
      values = c("highlight" = "blue", "normal" = "#CC0033", "different" = "green")
    ) +
    ggplot2::scale_y_continuous(
      name = y_axis_name,
      limits = y_limits,
      breaks = y_breaks
    ) +
    ggplot2::labs(x = x_label, y = y_label) +
    ggplot2::theme_minimal() +
    ggplot2::theme(
      plot.title = ggplot2::element_text(face = "bold", size = 14),
      plot.subtitle = ggplot2::element_text(size = 10, color = "gray40"),
      axis.title.y.left = ggplot2::element_text(color = "#CC0033", face = "bold", size = 16),
      axis.title.x = ggplot2::element_text(color = "#CC0033", face = "bold", size = 16),
      axis.text.x = ggplot2::element_text(angle = 45, hjust = 1, color = "black", size = 12),
      axis.text.y.left = ggplot2::element_text(color = "black", size = 12),
      panel.grid.major = ggplot2::element_line(linetype = "dashed", color = "gray80"),
      axis.line.x.bottom = ggplot2::element_line(color = "black"),
      axis.line.y.left = ggplot2::element_line(color = "black"),
      axis.text.y.right = ggplot2::element_blank(),
      axis.title.y.right = ggplot2::element_blank(),
      panel.grid.minor = ggplot2::element_blank(),
      legend.position = legend_position,
      legend.title = ggplot2::element_blank(),
      legend.text = ggplot2::element_text(face = "bold", size = 14)
     # ggbreak.axis.ticks.length = grid::unit(0, "pt"),
      #ggbreak.axis.line.size = 0
    )
  #Add the y_axis break to the plot
  add_y_breaks(p, scale_y_breaks)
  return(p)
}

# 8: Function that adds baseline to each dataset to form joined dataset with age,sex, BMI, & week day
add_baseline_function <- function(df, date_col, df_baseline, df_id, df_baseline_id)
{
  df$weekday <-  lubridate::wday(df[[date_col]], label = TRUE)
  df_w_baseline <- dplyr::inner_join(df, df_baseline, by = setNames(df_baseline_id, df_id))
  
  return(df_w_baseline)
}

# 9: Function for extracting Model 1 to 5 labels. It is needed by function 10
normalize_model_name <- function(x) {
  out <- stringr::str_extract(x, "Model_[0-9]+")
  ifelse(is.na(out), x, out)
}

# 10: Combine model results into a single table
make_model_summary_table <- function(model_list,
                                     col_name ="results") {

  if (is.null(names(model_list)) || any(names(model_list) == "")) {
    stop("`model_list` must be a *named* list (names are the model names).")
  }

  col_name <- as.character(col_name)
  
  purrr::imap_dfr(model_list, function(model_results, raw_model_name) {
    
    model_name <- normalize_model_name(raw_model_name)

    df <- model_results$lmer_model_results

     # Remove intercept and take the next row
    df_no_int <- dplyr::filter(df, term != "(Intercept)")

    if (nrow(df_no_int) == 0) {
      return(tibble::tibble(
        model = model_name,
        !!result_col := NA_character_
      ))
    }

    row <- df_no_int[1, , drop = FALSE]  # "Pick next row after intercept"

    est   <- round_for_model_4_and_5_b(row$estimate,  model_name)
    low   <- round_for_model_4_and_5_b(row$lower_ci,  model_name)
    high  <- round_for_model_4_and_5_b(row$upper_ci,  model_name)

    p_txt <- ifelse(row$p.value < 0.001, "<0.001", sprintf("%.3f", row$p.value))

    tibble::tibble(
      model = model_name,
      !!col_name := sprintf("%s [%s - %s]; p=%s", est, low, high, p_txt)
    )
  })
}
```

### Set up - read in datasets

```
# Read in  pain  data
pain <- read.csv(file = "data/PainArani2.csv")

# Read in step count data
step_data <- read.csv(file = "data/daily_steps.csv")

# Read in demographic data file
baseline_df <- read_excel("data/Baseline questionnaire in correct units.xlsx")

# Read in the KOOS pain and KOOS ADL scores data
koos <- read_excel("data/KOOS2012.xls", sheet ="KOOS_Clean")
```

### Data preparation prior to analysis

```
# 1: Data preparation for the pain and step count
# 1a: Filter out pain data for afternoon and evening pain only
pain_data <- pain[pain$notification_type %in% c('PAIN_OVERALL_MORNING', 'PAIN_OVERALL_AFTERNOON'), ]

# 1b: convert timestamp.Day in pain data and date in step count data to datetime.
pain_data$timestamp.Day <- as_datetime(pain_data$timestamp.Day)
step_data$date <- as_datetime(step_data$date)

# 1c: Join the pain and step datasets together by id and date, retaining only rows where the join conditions are non-NA and match in both tables.The inner_join result was checked to ensure correct behavior and that relevant observations were retained.

# Create the join condition
join_condition <- join_by(KOALAP.Name == user_id, timestamp.Day == date)
# Join them 
pain_and_step_df <- inner_join(pain_data, step_data, join_condition)

# 1d: Ensure each participant's data is within 90 days study period

# Initializing an empty dataframe to hold each user's censored data
pain_step_90days_df <- data.frame()

# All valid participants were onboarded. 
# Hard coding the onboarding time points since it varies
onboard_1 <- c(13, 14, 15, 19)
onboard_2 <- c(3, 7, 8, 9, 21, 25, 27)
onboard_3 <- c(16, 17, 18, 20, 23, 26, 28, 34, 35)
onboard_4 <- c(10, 30)
onboard_5 <- c(37, 38, 42)
onboard_6 <- c(5)

# The loop goes through user_id by ID and takes data from day of onboarding until day 90.
for (id in levels(factor(pain_and_step_df$user_id)))
{
  id_data <- pain_and_step_df[pain_and_step_df$user_id == id, ]
  
  if(id %in% onboard_1){
    subset_data <- id_data[id_data $timestamp.Day 
          <= (as.Date("2017-09-12") + days(90)) 
          & id_data$timestamp.Day > as.Date("2017-09-12"), ]
    
    subset_data$day_num <- as.numeric(as.Date(subset_data$timestamp.Day)
                                      - as.Date("2017-09-13")) + 1
    
  }else if (id %in% onboard_2) {
    subset_data <- id_data[id_data $timestamp.Day 
          <= (as.Date("2017-09-13") + days(90)) 
          & id_data$timestamp.Day > as.Date("2017-09-13"), ]
    
    subset_data$day_num <- as.numeric(as.Date(subset_data$timestamp.Day)
                                      - as.Date("2017-09-14")) + 1
    
  }else if (id %in% onboard_3) {
    subset_data <- id_data[id_data $timestamp.Day 
          <= (as.Date("2017-09-14") + days(90)) 
          & id_data$timestamp.Day > as.Date("2017-09-14"), ]
    
    subset_data$day_num <- as.numeric(as.Date(subset_data$timestamp.Day)
                                      - as.Date("2017-09-15")) + 1
  }else if (id %in% onboard_4) {
    subset_data <- id_data[id_data $timestamp.Day 
          <= (as.Date("2017-09-20") + days(90)) 
          & id_data$timestamp.Day > as.Date("2017-09-20"), ]
    
    subset_data$day_num <- as.numeric(as.Date(subset_data$timestamp.Day)
                                      - as.Date("2017-09-21")) + 1
    
  }else if (id %in% onboard_5) {
    subset_data <- id_data[id_data $timestamp.Day 
          <= (as.Date("2017-09-22") + days(90)) 
          & id_data$timestamp.Day > as.Date("2017-09-22"), ]
    
    subset_data$day_num <- as.numeric(as.Date(subset_data$timestamp.Day)
                                      - as.Date("2017-09-23")) + 1
  } else if (id %in% onboard_6) {
    subset_data <- id_data[id_data $timestamp.Day 
          <= (as.Date("2017-10-14") + days(90)) 
          & id_data$timestamp.Day > as.Date("2017-10-14"), ]
    
    subset_data$day_num <- as.numeric(as.Date(subset_data$timestamp.Day)
                                      - as.Date("2017-10-15")) + 1
  }else{
    next  # Skip not on the onboarded list.
  }
  
  # Adds the subset data for each participant to the dataframe
  pain_step_90days_df <- rbind(pain_step_90days_df, subset_data)

}

# 1e: Check for duplicated data and remove them.

# Create a new dataframe to hold the data to compute the duplicates
copy_90days_df <- pain_step_90days_df

# Sort data by user_id
copy_90days_df_sorted <- copy_90days_df[order(copy_90days_df$user_id), ]

# Check if there are more than one recording for the same notification and on the same day for users
duplicates_in_90days_df <- copy_90days_df_sorted[duplicated(copy_90days_df_sorted[, 
                          c("user_id", "timestamp.Day", "notification_type")]) | 
                          duplicated(copy_90days_df_sorted[, c("user_id", "timestamp.Day", "notification_type")], fromLast = TRUE), ]

# Print the duplicates (uncomment next print code to print out duplicates)
 # -Nine participants(user_id) each had one duplicated pain score for the same day (timestamp.Day) and notification type (notification_type)

#print(duplicates_in_90days_df[, c("user_id", "timestamp.Day", "timestamp.Time", "pain", "steps","notification_type", "day_num")])

# Remove the duplicates
 # - Eight of the nine participants with duplicated pain scores had two identical pain scores recorded on the same day for the same notification type; only the first record was retained. The ninth participants also had two pain scores recorded on the same day for the same notification_type, but only the first was kept because it fell within the valid response window, whereas the second did not.

unique_90days_df <- copy_90days_df_sorted[!duplicated(copy_90days_df_sorted[, 
                                 c("user_id", "timestamp.Day", "notification_type")]), ]

unique_90days_df <- unique_90days_df[, c("user_id", "timestamp.Day", "pain", "steps","notification_type", "day_num")]


# 1f: Computes the average of afternoon and evening pain if both present, take what is available if only one of them is present
avg_pain_step_90days <- unique_90days_df %>%
  group_by(user_id, timestamp.Day) %>%
  summarise(avg_pain = mean(pain), steps = unique(steps), 
            day_num = unique(day_num), .groups = "drop")

# 1g: Computes the lagged and changed values for pain and steps 
lag_avg_pain_step_90days <- lagging_function(avg_pain_step_90days, "avg_pain")
change_avg_pain_step_90days <- change_function(avg_pain_step_90days, "avg_pain")

# 2: Data preparation for the Demographic data

# 2a: Convert height into meters
df_baseline <- baseline_df %>%
  separate(Height, into = c("feet", "inch"), sep = "\\s*ft\\s*", convert = TRUE, remove = FALSE) %>%
  mutate(
    Height_m = (feet * 12 + ifelse(is.na(inch), 0, inch)) * 0.0254,
    Weight_kg = as.numeric(str_remove_all(Weight, "kg|\\s"))
  )

# 2b: Compute BMI using weight and height
df_baseline1 <- df_baseline[c("Koalap ID","Age","Gender","Weight_kg","Height_m")]
df_baseline1$BMI <-  df_baseline1$Weight_kg/(df_baseline1$Height_m^2)

# 2c: Changing userid 29 & 5 to 5 to match user5 in the pain and step dataset
df_baseline1 <- df_baseline1 %>%
  mutate(`Koalap ID` = if_else(`Koalap ID` == "29 & 5", "5", `Koalap ID`))

# 3: Data preparation for the KOOS
# 3a: Compute the median and IQR for the monthly scores
koos_summary <- koos %>%
  dplyr::select(contains("Subscale")) %>%
  pivot_longer(cols = everything(),
               names_to = "variable",
               values_to = "value") %>%
  summarise(
      median = round(median(value, na.rm = TRUE),1),
      p25 = round(quantile(value, 0.25, na.rm =TRUE),1),
      p75 = round(quantile(value, 0.75, na.rm = TRUE),1),
      .by = variable
    ) %>%
  mutate(result = paste0(median, " [", p25, " – ", p75, "]"))

# 4: Variables to build demographic table

age_qs <- quantile(df_baseline1$Age, probs = c(0.25, 0.5, 0.75), na.rm = TRUE)
bmi_qs <- quantile(df_baseline1$BMI, probs = c(0.25, 0.5, 0.75), na.rm = TRUE)
# Median, 25th percentile, # 75th percentile
Age_val <- sprintf("%d [%d–%d]", age_qs[2], age_qs[1], age_qs[3])
# Median, 25th percentile, # 75th percentile
BMI_val <- sprintf("%d [%d–%d]", round(bmi_qs[2]), round(bmi_qs[1]), round(bmi_qs[3]))
# Female proportion
n_female <- sum(df_baseline1$Gender == "F", na.rm = TRUE)
n_total <- sum(!is.na(df_baseline1$Gender))
female_val <- sprintf("%d (%.1f%%)", n_female, 100 * n_female / n_total)
KOOS_pain <- koos_summary[koos_summary$variable == "Oct_Pain_Subscale",]$result
KOOS_ADL <- koos_summary[koos_summary$variable == "Oct_ADL_Subscale",]$result
```

## Manuscript results

### Baseline demographic table

```
#Table_1 = Demographic characteristics table
Table_1 <- data.frame(
  Variable = c(
    "Age (years)",
    "BMI (kg/m^2)",
    "Female, n (%)",
    "KOOS, Pain ",
    "KOOS, ADL"
  ),
  Result = c(Age_val, BMI_val, female_val, KOOS_pain, KOOS_ADL)) 

knitr::kable(Table_1)
```

| Variable | Result |
| --- | --- |
| Age (years) | 64 [59–69] |
| BMI (kg/m^2) | 27 [25–34] |
| Female, n (%) | 13 (50.0%) |
| KOOS, Pain | 48.6 [41 – 59] |
| KOOS, ADL | 55.9 [42.3 – 68.4] |

### Summary statistics in result section

```
#------------------------
cat("No of participants in analysis:", " current day = ", length(unique((avg_pain_step_90days$user_id))), "; Lagged day = ", length(unique((lag_avg_pain_step_90days$user_id))), "; Changed day = ", length(unique((change_avg_pain_step_90days$user_id))))
```

```
## No of participants in analysis:  current day =  26 ; Lagged day =  26 ; Changed day =  26
```

```
#------------------------
no_records_per_user_lag <- count_function(lag_avg_pain_step_90days, "lagged_pain")
no_records_per_user_change <- count_function(change_avg_pain_step_90days, "change_in_steps")

cat("\nNo of records:", " current day = ", nrow(avg_pain_step_90days), "; Lagged day =",sum(no_records_per_user_lag$records_no), "; Changed day =", sum(no_records_per_user_change$records_no))
```

```
## 
## No of records:  current day =  1473 ; Lagged day = 1263 ; Changed day = 1263
```

```
#------------------------  
# Use the count function to determine no of participants with records >50days
no_records_per_user <- count_function(avg_pain_step_90days, "avg_pain")
cat("\nNo of participants with > 50 days record in current day analysis =", sum(no_records_per_user$records_no >50))
```

```
## 
## No of participants with > 50 days record in current day analysis = 18
```

```
#------------------------
```

### Day-to-day changes in pain and step count in people living with knee OA.

- Summary statistics for pain and step count

```
users_summary_stats <- get_users_summary_stats_function(avg_pain_step_90days, "avg_pain","steps")
# Print out summary stats for step count
cat("Median stepcount = ", round(median(users_summary_stats$median_step)), 
    "[IQR ", round(quantile(users_summary_stats$median_step, 0.25)), 
    " - ", round(quantile(users_summary_stats$median_step, 0.75)), "]", 
    " [range ", min(users_summary_stats$median_step), "-", 
    max(users_summary_stats$median_step), "] steps")
```

```
## Median stepcount =  3106 [IQR  1687  -  4433 ]  [range  423 - 7142 ] steps
```

```
# Print out summary stats for pain
cat("\nMedian pain = ", round(median(users_summary_stats$median_pain)), 
    "[IQR", round(quantile(users_summary_stats$median_pain, 0.25)), 
    " - ", round(quantile(users_summary_stats$median_pain, 0.75)), "]", 
    "[range ", min(users_summary_stats$median_pain), "-", 
    max(users_summary_stats$median_pain), "] NRS pain")
```

```
## 
## Median pain =  5 [IQR 2  -  7 ] [range  0 - 9 ] NRS pain
```

### Stepcount and pain distribution plot for each of the 26 participants

```
for (id in unique(avg_pain_step_90days$user_id)) {
  user_data <- avg_pain_step_90days[avg_pain_step_90days$user_id ==id, ]
  
  # Define step count scaling factor= (max steps/10)
  step_max <- max(user_data$steps, na.rm = TRUE)
  step_scale <- step_max / 10  # normalize to fit pain scale by divide by 10 
  

# Dual Y-axis Plot (pain and step), and same X-axis (Time points)
 each_user_step_pain_plot <- ggplot(user_data, aes(x = day_num)) +
    geom_line(aes(y = round(avg_pain), color = "Pain Score"), alpha = 0.8) +
    geom_point(aes(y = round(avg_pain), color = "Pain Score"), shape = 16) +
   
    scale_y_continuous(name = "Daily Pain Score (0-10)", limits = c(0, 10), breaks = seq(0, 10, by = 2),
                       sec.axis = sec_axis(~ . * step_scale, name = "Daily Step Count")) +
    
    scale_x_continuous(name = "Time Points (days)", limits = c(0, 90), 
                       breaks = seq(0, 90, by = 10)) +
    
    geom_line(aes(y = round(steps / step_scale), color = "Step Count"), linetype = "dashed", alpha = 0.8) +
    geom_point(aes(y = round(steps / step_scale), color = "Step Count"), shape = 17) +
    
    labs(x = "Time points (days)",
         subtitle = "Pain Score (solid) vs Step Count (dashed)") +
    
    scale_color_manual(values = c("Pain Score" = "#CC0033", "Step Count" = "#000066")) +
    
    theme_minimal(base_size = 14) +
    theme(
     # plot.title = element_text(face = "bold", size = 18),
      plot.subtitle = element_text(size = 16, color = "gray40"),
      axis.title.y.left = element_text(color = "#CC0033", face = "bold"),
      axis.title.y.right = element_text(color = "#000066", face = "bold"),
      axis.text.x = element_text(angle = 45, hjust = 1, size = 16),
      axis.text.y = element_text(size = 16),
      panel.grid.major = element_line(linetype = "dashed", color = "gray80"),
      legend.position = "top",
      legend.text = element_text(size = 12),
      legend.title = element_blank()
    ) 
  
  print(each_user_step_pain_plot)

  # Save all plots to output folder.
  #ggsave(filename = paste0("outputs/step_avgpain_plot/plot_user_",id, ".jpg"), plot = each_user_step_pain_plot, width = 12, height = 8, dpi = 300)
  
}
```

### Two illustrative examples of step count and pain plot: Figure 1 and Figure 2

- Figure 1: Daily pain and step count over time for Participant
  21.

- Figure
2: Daily pain and step count over time for Participant 30.

### Associations across the population (i.e., group) modelling with results

```
# A) Fitting an unadjusted model across participants association models

# Model 1: outcome = Current day Pain [day t]; independent variable = Current day step count [day t]
Model_1 <- across_id_lmer_function(avg_pain_step_90days, "avg_pain", "steps", "user_id", ci_method = "profile")

# Model 2: outcome = Current day Pain [day t]; independent variable = Prior day’s step count [day t-1]
Model_2 <- across_id_lmer_function(lag_avg_pain_step_90days, "avg_pain", "lagged_steps", "user_id", ci_method = "profile")

# Model 3: outcome = Current day Pain [day t]; independent variable = Change from prior day’s to the current day’s step count [(day t) – (day t-1)]
Model_3 <- across_id_lmer_function(change_avg_pain_step_90days, "avg_pain", "change_in_steps", "user_id", ci_method = "profile")

# Model 4: outcome = Current day’s step count [day t]; independent variable = Prior day’s pain [day t – 1]
Model_4 <- across_id_lmer_function(lag_avg_pain_step_90days, "steps", "lagged_pain", "user_id", ci_method = "profile")

# Model 5: outcome = Current day’s step count [day t]; independent variable = Change from prior day's to the current day’s pain [(day t) – (day t-1)]
Model_5 <- across_id_lmer_function(change_avg_pain_step_90days, "steps", "change_in_pain", "user_id", ci_method = "profile")

# B) Extract the estimates and effect sizes and their 95%CI for the models

# List all models so that it can be easily looped through
table_2_models <- c("Model_1", "Model_2", "Model_3", "Model_4", "Model_5")

model_list <- mget(table_2_models, envir = .GlobalEnv)

Table_2 <- imap_dfr(model_list, \(model_results, model_name) {
  model_results$lmer_model_results %>%                              
    filter(term != "(Intercept)") %>%
    transmute(
      Model = model_name,
      Est = round_for_model_4_and_5(estimate, model_name),
      Est_low_ci = round_for_model_4_and_5(lower_ci, model_name),
      Est_up_ci = round_for_model_4_and_5(upper_ci, model_name),
      Effect_size = smart_round_effect(effect_std_beta),
      Effect_size_low_ci = smart_round_effect(effect_std_beta_lo),
      Effect_size_up_ci = smart_round_effect(effect_std_beta_hi),
      p_value = ifelse(p.value < 0.001, "<0.001", sprintf("%.3f", p.value))
    )
})

# C) Show the formatted table for the association across the population
# print(Table_2)
knitr::kable(Table_2)
```

| Model | Est | Est\_low\_ci | Est\_up\_ci | Effect\_size | Effect\_size\_low\_ci | Effect\_size\_up\_ci | p\_value |
| --- | --- | --- | --- | --- | --- | --- | --- |
| Model\_1 | 0.04 | 0.01 | 0.06 | 0.05 | 0.02 | 0.08 | 0.002 |
| Model\_2 | 0.05 | 0.03 | 0.07 | 0.07 | 0.04 | 0.11 | <0.001 |
| Model\_3 | -0.01 | -0.03 | 0.01 | -0.01 | -0.04 | 0.02 | 0.544 |
| Model\_4 | -0.4 | -98 | 97 | -0.0002 | -0.05 | 0.05 | 0.994 |
| Model\_5 | -2 | -90 | 86 | -0.001 | -0.05 | 0.04 | 0.960 |

### Associations within individuals modeling

```
# within_id_Model_1: outcome = Current day Pain [day t]; independent variable = Current day step count [day t]
within_id_Model_1 <- within_users_ols_function(avg_pain_step_90days, "avg_pain", "steps")

# within_id_Model_2: outcome = Current day Pain [day t]; independent variable = Prior day’s step count [day t-1]
within_id_Model_2 <- within_users_ols_function(lag_avg_pain_step_90days, "avg_pain", "lagged_steps")


# within_id_Model_3: outcome = Current day Pain [day t]; independent variable = Change from prior day’s to the current day’s step count [(day t) – (day t-1)]
within_id_Model_3 <- within_users_ols_function(change_avg_pain_step_90days, "avg_pain", "change_in_steps")

# within_id_Model_4: outcome = Current day’s step count [day t]; independent variable = Prior day’s pain [day t – 1]
within_id_Model_4 <- within_users_ols_function(lag_avg_pain_step_90days, "steps", "lagged_pain")

# within_id_Model_5: outcome = Current day’s step count [day t]; independent variable = Change from prior day’s to the current day’s pain [(day t) – (day t-1)]
within_id_Model_5 <- within_users_ols_function(change_avg_pain_step_90days, "steps", "change_in_pain")
```

### Bidirectional association Figures 3 and 4 for participants and group

```
# 1) Putting within model result data frames into a named list
within_models <- list(
  M1 = within_id_Model_1,
  M2 = within_id_Model_2,
  M3 = within_id_Model_3,
  M4 = within_id_Model_4,
  M5 = within_id_Model_5
)

# 2) Clean + prefix columns, then join across models by user_id
within_id_all_models_wide <- imap(within_models, \(df, prefix) {
  df %>%
    filter(term != "(Intercept)") %>%
    transmute(
      user_id,
      !!paste0(prefix, "_Est")        := round(estimate, 3),
      !!paste0(prefix, "_Est_low_ci") := round(lower_ci, 3),
      !!paste0(prefix, "_Est_up_ci")  := round(upper_ci, 3)
    )
}) %>%
  reduce(full_join, by = "user_id")

within_id_all_models_wide$user_id <- as.character(within_id_all_models_wide$user_id)
# Ensure all within participant results are in type double
within_id_all_models_wide <- convert_to_dbl(within_id_all_models_wide)

# 3) Putting across model to be in the same format as within participant model
group_row <- Table_2 %>%
  dplyr::filter(Model %in% c("Model_1", "Model_2", "Model_3", "Model_4","Model_5")) %>%
  dplyr::mutate(prefix = recode(Model,
                         "Model_1" = "M1",
                         "Model_2" = "M2",
                         "Model_3" = "M3",
                         "Model_4" = "M4",
                         "Model_5" = "M5")) %>%
  dplyr::select(prefix, Est, Est_low_ci, Est_up_ci) %>%
  tidyr::pivot_wider(
    names_from = prefix,
    values_from = c(Est, Est_low_ci, Est_up_ci),
    names_glue = "{prefix}_{.value}"
  ) %>%
  dplyr::mutate(user_id = "Group") %>%
  dplyr::select(user_id, everything())

# 3a) Arranging the across model so that it is exactly as the within model table
# How I want it order
desired_order <- c(
  "user_id",
  as.vector(rbind(
    paste0("M", 1:5, "_Est"),
    paste0("M", 1:5, "_Est_low_ci"),
    paste0("M", 1:5, "_Est_up_ci")
  ))
)
# Ordering it
group_row <- group_row %>%
  dplyr::select(dplyr::any_of(desired_order))

# Ensure all group results are in type double
group_row <- convert_to_dbl(group_row)

# 4) Combine across and within participant model results
within_id_all_models_with_group <- dplyr::bind_rows(
  within_id_all_models_wide,
  group_row
) %>%
  dplyr::select(dplyr::any_of(desired_order))

#5) Plots the regression estimate across all analysis

## 5A) Plot for Model_1 to Model_5 without Y axis breaks. Results for model 1 is Figure 3. Model2-5 are supplementary ## Figures B1 to B4

# 5A_1) Plot for Model_1 with y-axis breaks: outcome = Current day Pain [day t]; independent variable = Current day step count [day t]
M1_plot_with_breaks <- make_ggbreak_ci_plot(
  df = within_id_all_models_with_group,
  est = "M1_Est",
  low_ci = "M1_Est_low_ci",
  up_ci = "M1_Est_up_ci",
  y_axis_name = "Pain [day t] estimate per 1000 increase in step count [day t]",  
  y_limits = c(-12, 2.2),
  y_breaks = 
   c(seq(-1, 1, by = 0.2), -10, -5, -4, -1.0, 1.0, 1.6, 1.9, 2.1),
  scale_y_breaks = list(
    list(range = c(2.0, 2.1), scales = 1),
    list(range = c(1.6, 1.9), scales = 5),
    list(range = c(1.0, 1.1), scales = 2),
    list(range = c(-4, -1.0), scales = 20),
    list(range = c(-10, -5), scales = 4.5)
  ),
  y_label = "Pain [day t] estimate per 1000 increase in step count [day t]"
)

# 5A_2) Plot for Model_2 with y-axis breaks: outcome = Current day Pain [day t]; independent variable = Prior day’s step count [day t-1]
M2_plot_with_breaks <- make_ggbreak_ci_plot(
  df = within_id_all_models_with_group,
  est = "M2_Est",
  low_ci = "M2_Est_low_ci",
  up_ci = "M2_Est_up_ci",
  y_axis_name = "M2) Pain est. per 1000 steps[day t - 1]",  
  y_limits = c(-41, 24),
  y_breaks = c(seq(-1, 1, by = 0.1), -40, -38, -1,-7, -8, 20, 22, 24),
  scale_y_breaks = list(
    list(range = c(1.0, 22), scales = 2),
    list(range = c(-7, -1.0), scales = 15),
    list(range = c(-38, -8), scales = 5)
  ),
  y_label = ""
)
# 5A_3) Plot for Model_3 with y-axis breaks: outcome = Current day Pain [day t]; independent variable = Change from prior day’s to the current day’s step count [(day t) – (day t-1)]
M3_plot_with_breaks <- make_ggbreak_ci_plot(
  df = within_id_all_models_with_group,
  est = "M3_Est",
  low_ci = "M3_Est_low_ci",
  up_ci = "M3_Est_up_ci",
  y_axis_name = "M3) Pain est. per 1000 steps[day t - (day t - 1)]",  
  y_limits = c(-96, 1),
  y_breaks = c(seq(-1, 1, by = 0.1), -96, -89,-70,-60,-56, -1, -7, -8, -10, -20),
  scale_y_breaks = list(
    list(range = c(-7, -1.0), scales = 20),
    list(range = c(-15, -8), scales = 5),
    list(range = c(-89, -56), scales = 5)
  ),
  y_label = ""
)
# 5A_4) Plot for Model_4 with y-axis breaks: outcome = Current day’s step count [day t]; independent variable = Prior day’s pain [day t – 1]
M4_plot_with_breaks <- make_ggbreak_ci_plot(
  df = within_id_all_models_with_group,
  est = "M4_Est",
  low_ci = "M4_Est_low_ci",
  up_ci = "M4_Est_up_ci",
  y_axis_name = "M4) Step est. per 1 unit pain[day t - 1]",  
  y_limits = c(-2000, 2000),
  y_breaks = seq(-2000, 2000, by = 200),
  scale_y_breaks = list(),
  y_label = ""
)
# 5A_5) Plot for Model_5 with y-axis breaks: outcome = Current day’s step count [day t]; independent variable = Change from prior day’s to the current day’s pain [(day t) – (day t-1)]
M5_plot_with_breaks <- make_ggbreak_ci_plot(
  df = within_id_all_models_with_group,
  est = "M5_Est",
  low_ci = "M5_Est_low_ci",
  up_ci = "M5_Est_up_ci",
  y_axis_name = "M5) Step est. per 1 unit pain[day t - (day t - 1)]",  
  y_limits = c(-2000, 2000),
  y_breaks = seq(-2000, 2000, by = 200),
  scale_y_breaks = list(),
  y_label = ""
)

## 5B) Plot for Model_2 to Model_5 without Y-axis breaks to create Figure 4
# 5B_1) Plot for Model_2: outcome = Current day Pain [day t]; independent variable = Prior day’s step count [day t-1]
M2_plot <- make_ordered_ci_plot(
  within_id_all_models_with_group,
  "M2_Est",
  "M2_Est_low_ci",
  "M2_Est_up_ci",
  "M2) Pain est. per 1000 steps[day t - 1]",
  c(-1.0, 1.0),
  seq(-1.0, 1.0, by = 0.1),
  "y_label",
  x_label = "Participant's IDs",
  highlight_ids = c("21", "30"),
  group_id = "Group",
  legend_position = "none"
)

# 5B_2) Plot for Model_3: outcome = Current day Pain [day t]; independent variable = Change from prior day’s to the current day’s step count [(day t) – (day t-1)]
M3_plot <- make_ordered_ci_plot(
  within_id_all_models_with_group,
  "M3_Est",
  "M3_Est_low_ci",
  "M3_Est_up_ci",
  "M3) Pain est. per 1000 steps[day t - (day t - 1)]",
  c(-1.0, 1.0),
  seq(-1.0, 1.0, by = 0.1),
  "y_label",
  x_label = "Participant's IDs",
  highlight_ids = c("21", "30"),
  group_id = "Group",
  legend_position = "none"
)

# 5B_3) Plot for Model_4: outcome = Current day’s step count [day t]; independent variable = Prior day’s pain [day t – 1]

M4_plot <- make_ordered_ci_plot(
  within_id_all_models_with_group,
  "M4_Est",
  "M4_Est_low_ci",
  "M4_Est_up_ci",
  "M4) Step est. per 1 unit pain[day t - 1]",
  c(-2000, 2000),
  seq(-2000, 2000, by = 200),
  "y_label",
  x_label = "Participant's IDs",
  highlight_ids = c("21", "30"),
  group_id = "Group",
  legend_position = "none"
)
# 5B_4) Plot for Model_5: outcome = Current day’s step count [day t]; independent variable = Change from prior day’s to the current day’s pain [(day t) – (day t-1)]

M5_plot <- make_ordered_ci_plot(
  within_id_all_models_with_group,
  "M5_Est",
  "M5_Est_low_ci",
  "M5_Est_up_ci",
  "M5) Step est. per 1 unit pain[day t - (day t - 1)]",
  c(-2000, 2000),
  seq(-2000, 2000, by = 200),
  "y_label",
  x_label = "Participant's IDs",
  highlight_ids = c("21", "30"),
  group_id = "Group",
  legend_position = "none"
)
```

- Figure 3: Association between pain and step count [per 1000 unit
  increases in step count] on the current day [day t] for each participant
  plus whole population [“Group”] estimate
- Figure 4: Association between pain and step count [per 1000 unit
  increase in step count] or step count and pain [per 1 unit increase in
  NRS pain] for each participant plus whole population [“Group”] estimate

## Sensitivity analysis

### 1) Results for stepcount[day t] and pain[day t] modelled using different pain definitions

- Model\_1 gives the unadjusted estimate for pain[day t] for 1000
  increase in stepcount[day t]
- The pain value in the Model\_1 = mean of the afternoon and evening
  pain if both were available for that day. If one of either afternoon or
  evening pain was missing, the available pain score was used.
- afternoon\_pain = pain score taken in the afternoon
- evening pain = pain score taken in the evening
- avg\_aftnoon\_evening\_pain = mean of the afternoon and evening pain
  when both are available for that day

```
# Create seperate dataframe (df) for the different pain types
afternoon_pain_df <- unique_90days_df[unique_90days_df$notification_type == "PAIN_OVERALL_MORNING", ]

evening_pain_df <- unique_90days_df[unique_90days_df$notification_type == "PAIN_OVERALL_AFTERNOON", ]

#Get average of the two pains when both are available
avg_aftnoon_evening_pain_df <- unique_90days_df %>%
  group_by(user_id, timestamp.Day) %>%
  # The filter helps ensure the mean is taken only when exactly two pain notifications exist
  filter(all(c("PAIN_OVERALL_MORNING", "PAIN_OVERALL_AFTERNOON") %in% notification_type)) %>%
  summarise(mean_pain = mean(pain),
            steps = unique(steps), 
            day_num = unique(day_num),
            timestamp.Day = unique(timestamp.Day), .groups = "drop")

# Modeling using only afternoon pain
afternoon_pain_model <- across_id_lmer_function(afternoon_pain_df, "pain", "steps", "user_id", ci_method = "profile")
# Modeling using only evening pain
evening_pain_model <- across_id_lmer_function(evening_pain_df, "pain", "steps", "user_id", ci_method = "profile")
avg_aftnoon_evening_pain <- across_id_lmer_function(avg_aftnoon_evening_pain_df, "mean_pain", "steps", "user_id", ci_method = "profile")


# List all models so that it can be easily looped through
pain_models <- c("Model_1", "afternoon_pain_model", "evening_pain_model", "avg_aftnoon_evening_pain")

pain_model_list <- mget(pain_models, envir = .GlobalEnv)

Sup_Table_1 <- imap_dfr(pain_model_list, \(model_results, model_name) {
  model_results$lmer_model_results %>%                               # obj$lmer_model_results 
    filter(term != "(Intercept)") %>%
    transmute(
      `Outcome = pain[day t]; predictor stepcount[day t]` = model_name,
      Est = round_for_model_4_and_5_b(estimate, model_name),
      Est_low_ci = round_for_model_4_and_5_b(lower_ci, model_name),
      Est_up_ci = round_for_model_4_and_5_b(upper_ci, model_name),
      p_value = ifelse(p.value < 0.001, "<0.001", sprintf("%.3f", p.value))
    )
})

# Print the table
knitr::kable(Sup_Table_1)
```

| Outcome = pain[day t]; predictor stepcount[day t] | Est | Est\_low\_ci | Est\_up\_ci | p\_value |
| --- | --- | --- | --- | --- |
| Model\_1 | 0.036 | 0.013 | 0.058 | 0.002 |
| afternoon\_pain\_model | 0.024 | -0.003 | 0.050 | 0.077 |
| evening\_pain\_model | 0.044 | 0.018 | 0.069 | <0.001 |
| avg\_aftnoon\_evening\_pain | 0.028 | 0.004 | 0.052 | 0.021 |

### 2) Results for stepcount[day t] and pain[day t] modelled using data based on user’s observation level

- Model\_1 gives the unadjusted estimate for pain[day t] for 1000
  increase in stepcount[day t] (Outcome = pain[day t]; predictor =
  stepcount[day t])
- The pain value in the Model\_1 = mean of the afternoon and evening
  pain if both were available for that day. If one of either afternoon or
  evening pain was missing, the available pain score was used.
- above\_20\_pct\_obs … = data of participants with >20% of the 90
  required observations
- above\_50\_pct\_obs … = data of participants with >50% of the 90
  required observations

```
# Defining the data to use first
# The maximum observation count per participant is 90, reflecting the 90-day monitoring period.

# Filtering out data for participants with more than 20% of the observations. 
above_20_pct_obs_df <- avg_pain_step_90days %>%
  group_by(user_id) %>%
  filter(n() >18) %>%
  ungroup()

# Filtering out data for participants with more than 20% of the observations. 
above_50_pct_obs_df <- avg_pain_step_90days %>%
  group_by(user_id) %>%
  filter(n() >45) %>%
  ungroup()

# Modelling the observation data
above_20_pct_obs_model <- across_id_lmer_function(above_20_pct_obs_df, "avg_pain", "steps", "user_id", ci_method = "profile")
above_50_pct_obs_model <- across_id_lmer_function(above_50_pct_obs_df, "avg_pain", "steps", "user_id", ci_method = "profile")

# List all models so that it can be easily looped through
# Model 1 is the model presented in the manuscript's Table 2.
pct_obs_models <- c("Model_1","above_20_pct_obs_model", "above_50_pct_obs_model")

pct_obs_models_list <- mget(pct_obs_models, envir = .GlobalEnv)

Supl_Table_2 <- imap_dfr(pct_obs_models_list, \(model_results, model_name) {
  model_results$lmer_model_results %>%                                
    filter(term != "(Intercept)") %>%
    transmute(
      Models = model_name,
      Est = round_for_model_4_and_5_b(estimate, model_name),
      Est_low_ci = round_for_model_4_and_5_b(lower_ci, model_name),
      Est_up_ci = round_for_model_4_and_5_b(upper_ci, model_name),
      p_value = ifelse(p.value < 0.001, "<0.001", sprintf("%.3f", p.value))
    )
})

# Create a table that holds count of participants based on the observation levels
no_in_obs_df <- data.frame(
  Models = c("Model_1", "above_20_pct_obs_model", "above_50_pct_obs_model"),
  Participants_included = c(length(unique(avg_pain_step_90days$user_id)),length(unique(above_20_pct_obs_df$user_id)),length(unique(above_50_pct_obs_df$user_id)))
)
# Create a table of participant counts and model results for each observation level.
Sup_Table_2 <- inner_join(Supl_Table_2, no_in_obs_df, by = "Models") %>%
  dplyr::select(Models, Participants_included, everything())

# print the table
knitr::kable(Sup_Table_2)
```

| Models | Participants\_included | Est | Est\_low\_ci | Est\_up\_ci | p\_value |
| --- | --- | --- | --- | --- | --- |
| Model\_1 | 26 | 0.036 | 0.013 | 0.058 | 0.002 |
| above\_20\_pct\_obs\_model | 24 | 0.035 | 0.013 | 0.057 | 0.002 |
| above\_50\_pct\_obs\_model | 18 | 0.040 | 0.016 | 0.063 | 0.001 |

### 3) Bidrectional association models after adjusting for Age, Sex, BMI and Day of week

```
# 1) Adding age, sex, BMI, day of week to the current day, lagged day and change day datasets

# 1a) Updating the id in the baseline to match as same datatype as in the other datasets
df_baseline1$`Koalap ID` <- as.integer(df_baseline1$`Koalap ID`)
df_baseline1$Gender <- as.factor(df_baseline1$Gender)

# 1b) Add age,sex,BMI,day of week to the pain and step dataframe
pain_step_w_baseline_df <- add_baseline_function(df = avg_pain_step_90days, date_col ="timestamp.Day",
                      df_baseline = df_baseline1, df_id = "user_id", 
                      df_baseline_id = "Koalap ID")
# 1c) Add age,sex,BMI, day of week to the lagged (pain and step) dataframe
lagged_data_w_baseline_df <- add_baseline_function(df = lag_avg_pain_step_90days, date_col ="timestamp.Day",
                      df_baseline = df_baseline1, df_id = "user_id", 
                      df_baseline_id = "Koalap ID")
# 1d) Add age,sex,BMI, day of week to the lagged (pain and step) dataframe
changed_data_w_baseline_df <- add_baseline_function(df = change_avg_pain_step_90days, date_col ="timestamp.Day",
                      df_baseline = df_baseline1, df_id = "user_id", 
                      df_baseline_id = "Koalap ID")

# 2) Adjusting all models for covariates
# Model 1 adjusted: outcome = Current day Pain [day t]; independent variable = Current day step count [day t]
  # Adjusted for age, sex, BMI
Model_1_adj_1 <- across_id_lmer_function(pain_step_w_baseline_df, "avg_pain", c("steps", "Age","Gender","BMI"), "user_id", ci_method = "profile")
  # Adjusted for  Age,Sex, BMI and day of week
Model_1_adj_2 <- across_id_lmer_function(pain_step_w_baseline_df, "avg_pain", c("steps", "Age","Gender","BMI","weekday"), "user_id", ci_method = "profile")

# Model 2 adjusted: outcome = Current day Pain [day t]; independent variable = Prior day’s step count [day t-1]
  # Adjusted for age, sex, BMI
Model_2_adj_1 <- across_id_lmer_function(lagged_data_w_baseline_df, "avg_pain", c("lagged_steps", "Age","Gender","BMI"), "user_id", ci_method = "profile")
  # Adjusted for age,sex, BMi and day of week
Model_2_adj_2 <- across_id_lmer_function(lagged_data_w_baseline_df, "avg_pain", c("lagged_steps","Age","Gender","BMI","weekday"), "user_id", ci_method = "profile")

# Model 3: outcome = Current day Pain [day t]; independent variable = Change from prior day’s to the current day’s step count [(day t) – (day t-1)]
  # Adjusted for age, sex, BMI
Model_3_adj_1 <- across_id_lmer_function(changed_data_w_baseline_df, "avg_pain", c("change_in_steps","Age","Gender","BMI"), "user_id", ci_method = "profile")
  # Adjusted for  age,sex, BMI and day of week
Model_3_adj_2 <- across_id_lmer_function(changed_data_w_baseline_df, "avg_pain", c("change_in_steps","Age","Gender","BMI","weekday"), "user_id", ci_method = "profile")

# Model 4: outcome = Current day’s step count [day t]; independent variable = Prior day’s pain [day t – 1]
  # Adjusted for age, sex, BMI
Model_4_adj_1 <- across_id_lmer_function(lagged_data_w_baseline_df, "steps", c("lagged_pain","Age","Gender","BMI"), "user_id", ci_method = "profile")
  # Adjusted for age, sex, BMI and day of week
Model_4_adj_2 <- across_id_lmer_function(lagged_data_w_baseline_df, "steps", c("lagged_pain","Age","Gender","BMI","weekday"),"user_id", ci_method = "profile")

# Model 5: outcome = Current day’s step count [day t]; independent variable = Change from prior day’s to the current day’s pain [(day t) – (day t-1)]
  # Adjusted for age, sex, BMI
Model_5_adj_1 <- across_id_lmer_function(changed_data_w_baseline_df, "steps", c("change_in_pain", "Age","Gender","BMI"),"user_id", ci_method = "profile")
  # Adjusted for age, aex, BMI and day of week
Model_5_adj_2 <- across_id_lmer_function(changed_data_w_baseline_df, "steps", c("change_in_pain","Age","Gender","BMI","weekday"), "user_id", ci_method = "profile")


#3) Combine all adjusted models and print the results

# Model_lists needed by the function 
unadjusted_models <- c("Model_1","Model_2","Model_3","Model_4","Model_5")
model_list_1 <- mget(unadjusted_models, envir = .GlobalEnv)

sup_table_a <- make_model_summary_table(model_list_1,col_name = "Est[95%CI]; P_val (Unadjusted)")

adjusted_models_1 <- c("Model_1_adj_1","Model_2_adj_1","Model_3_adj_1","Model_4_adj_1","Model_5_adj_1")
model_list_2 <- mget(adjusted_models_1, envir = .GlobalEnv)

sup_table_b <- make_model_summary_table(model_list_2,col_name = "Est[95%CI]; P_val (Age_Sex_BMI_Adjusted)")

adjusted_models_2 <- c("Model_1_adj_2","Model_2_adj_2","Model_3_adj_2","Model_4_adj_2","Model_5_adj_2")
model_list_3 <- mget(adjusted_models_2, envir = .GlobalEnv)

sup_table_c <- make_model_summary_table(model_list_3, col_name = "Est[95%CI]; P_val (Age_Sex_BMI_DayofWeek_Adjusted)")

# Join all model tables together
# Note: the inner_join result was checked to ensure correct behavior and that relevant observations were retained.
Sup_Table_3 <- sup_table_a %>%
  dplyr::inner_join(sup_table_b, by = "model") %>%
  dplyr::inner_join(sup_table_c, by = "model")

# Print the table
knitr::kable(Sup_Table_3)
```

| model | Est[95%CI]; P\_val (Unadjusted) | Est[95%CI]; P\_val (Age\_Sex\_BMI\_Adjusted) | Est[95%CI]; P\_val (Age\_Sex\_BMI\_DayofWeek\_Adjusted) |
| --- | --- | --- | --- |
| Model\_1 | 0.036 [0.013 - 0.058]; p=0.002 | 0.036 [0.013 - 0.058]; p=0.002 | 0.033 [0.010 - 0.055]; p=0.004 |
| Model\_2 | 0.050 [0.027 - 0.074]; p=<0.001 | 0.050 [0.026 - 0.074]; p=<0.001 | 0.048 [0.024 - 0.071]; p=<0.001 |
| Model\_3 | -0.006 [-0.027 - 0.014]; p=0.544 | -0.006 [-0.026 - 0.014]; p=0.545 | -0.007 [-0.027 - 0.014]; p=0.526 |
| Model\_4 | -0.4 [-98 - 97]; p=0.994 | -1 [-99 - 96]; p=0.983 | 11 [-85 - 108]; p=0.816 |
| Model\_5 | -2 [-90 - 86]; p=0.960 | -2 [-90 - 87]; p=0.970 | -17 [-104 - 71]; p=0.711 |
