# Supplementary material for: Exploring the bidirectional temporal association between daily knee pain and physical activity in people with knee osteoarthritis: An exploratory smartwatch study
Source: Osteoarthr Cartil Open. 2026 Jan 31;8(1):100753. doi: 10.1016/j.ocarto.2026.100753 (PMC12907850; doi:10.1016/j.ocarto.2026.100753)
Supplement: Multimedia component 3 [file mmc3.docx]

**Supplementary file 3 to the article** “Exploring the bidirectional temporal association between daily knee pain and physical activity in people with knee osteoarthritis: an exploratory smartwatch study.”

Table 1: Association between current-day pain and step count across all 26 participants using different pain definitions

| Models | Outcome | Independent Variable | Estimate  [95% CI] | P-value |
| --- | --- | --- | --- | --- |
| Model 1 | Pain [day t] | Step count [day t] | 0.036 [0.013 to 0.058] | 0.002* |
| Afternoon model | Afternoon pain [day t] | Step count [day t] | 0.024 [-0.003 to 0.050] | 0.077 |
| Evening model | Evening pain [day t] | Step count [day t] | 0.044[0.018 to 0.069] | <0.001* |
| Average model | Average pain [day t] | Step count [day t] | 0.028[0.004 to 0.052] | 0.021* |

Associations reported are per 1000 unit increase in step count; ‘day t’ represents current day.

**Model 1** is the model reported in Table 1 of the article and was fitted using pain defined as the mean of the afternoon and evening pain scores when both were available for a given day; when only one pain score was available, that value was used. The **afternoon model** was fitted using only pain scores collected in the afternoon. The **evening model** was fitted using only pain scores collected in the evening. The **average model** was fitted using the mean of the afternoon and evening pain scores only when both were available for that day. * Indicates statistical significance at p < 0.005.
