# Supplementary material for: Exploring the bidirectional temporal association between daily knee pain and physical activity in people with knee osteoarthritis: An exploratory smartwatch study
Source: Osteoarthr Cartil Open. 2026 Jan 31;8(1):100753. doi: 10.1016/j.ocarto.2026.100753 (PMC12907850; doi:10.1016/j.ocarto.2026.100753)
Supplement: Multimedia component 4 [file mmc4.docx]

**Supplementary file 4 to the article** “Exploring the bidirectional temporal association between daily knee pain and physical activity in people with knee osteoarthritis: an exploratory smartwatch study.”

Table 1: Association between current-day pain and step count across all participants using different observation levels

| Models | Participants  Included | Outcome | Independent Variable | Estimate  [95% CI] | P-value |
| --- | --- | --- | --- | --- | --- |
| Model 1 | 26 | Pain [day t] | Step count [day t] | 0.036 [0.013 to 0.058] | 0.002* |
| >20% model | 24 | Pain [day t] | Step count [day t] | 0.035 [0.013 to 0.057] | 0.002* |
| >50% model | 18 | Pain [day t] | Step count [day t] | 0.040 [0.016 to 0.063] | 0.001* |

Associations reported are per 1000 unit increase in step count; ‘day t’ represents current day.

**Pain** was defined as the mean of the afternoon and evening pain scores when both were available for a given day; when only one pain score was available, that value was used.

**Model 1** corresponds to the model reported in Table 1 of the article and was fitted using data for all participants regardless of their level of observation. The **>20% model** was fitted using data restricted to participants with more than 20% of expected observations available. The **>50% model** was fitted using data restricted to participants with more than 50% of expected observations available. * Indicates statistical significance at p < 0.005.
