# Supplementary material for: Exploring the bidirectional temporal association between daily knee pain and physical activity in people with knee osteoarthritis: An exploratory smartwatch study
Source: Osteoarthr Cartil Open. 2026 Jan 31;8(1):100753. doi: 10.1016/j.ocarto.2026.100753 (PMC12907850; doi:10.1016/j.ocarto.2026.100753)
Supplement: Multimedia component 5 [file mmc5.docx]

**Supplementary file 5 to the article** “Exploring the bidirectional temporal association between daily knee pain and physical activity in people with knee osteoarthritis: an exploratory smartwatch study.”

Table 1: Associations between step count and pain, or pain and step count across all 26 participants: unadjusted and adjusted models

| Models | Outcome | Independent Variable | Estimate [95% CI]; p  (Unadjusted) | Estimate [95% CI]; p  (Age, Sex, BMI adjusted) | Estimate [95% CI]; p  (Age, Sex, BMI, Weekday adjusted) |
| --- | --- | --- | --- | --- | --- |
| Model 1 | Pain [day t] | Step count [day t] | 0.036 [0.013 to 0.058]; p=0.002* | 0.036 [0.013 to 0.058]; p=0.002* | 0.033 [0.010 to 0.055]; p=0.004* |
| Model 2 | Pain [day t] | Step count [day t-1] | 0.050 [0.027 to 0.074]; p=<0.001* | 0.050 [0.026 to 0.074]; p=<0.001* | 0.048 [0.024 to 0.071]; p=<0.001* |
| Model 3 | Pain [day t] | Step count [(day t) – (day t-1)] | -0.006 [-0.027 to 0.014]; p=0.544 | -0.006 [-0.026 -to 0.014]; p=0.545 | -0.007 [-0.027 to 0.014]; p=0.526 |
| Model 4 | step count [day t] | pain [day t – 1] | -0.4 [-98 to 97]; p=0.994 | -1 [-99 to 96]; p=0.983 | 11 [-85 to 108]; p=0.816 |
| Model 5 | step count [day t] | pain [(day t) – (day t-1)] | -2 [-90 to 86]; p=0.960 | -2 [-90 to 87]; p=0.970 | -17 [-104 to 71]; p=0.711 |

Associations reported are per 1000-step increase or per one-unit increase in NRS pain, depending on the analysis; ‘day t’ represents current day; ‘[day t-1]’ represents prior day; ‘[(day t) – (day t-1)]’ represents change from prior day to the current day; BMI: Body Mass Index; CI: Confidence Interval; p: probability value. The unadjusted estimate corresponds to the results presented for Model 1-5 in Table 1 of the article.

* Indicates statistical significance at p < 0.005.
